# Assessing Clinical Competence of Postgraduate Dental Specialty Trainees: A Scoping Review

**DOI:** 10.1111/eje.70060

**Published:** 2025-10-23

**Authors:** Fatemeh Amir‐Rad, Susan Morison, Sabarinath Prasad, Nabil Zary, Gerry McKenna

**Affiliations:** ^1^ Hamdan Bin Mohammed College of Dental Medicine Mohammed Bin Rashid University of Medicine and Health Sciences, Dubai Health Dubai UAE; ^2^ School of Medicine, Dentistry and Biomedical Sciences Queen's University Belfast Belfast UK; ^3^ Institute for Excellence in Health Professions Education Mohammed Bin Rashid University of Medicine and Health Sciences, Dubai Health Dubai UAE

**Keywords:** assessment, clinical competence, dental specialty training, postgraduate, scoping review

## Abstract

**Background:**

Assessment is essential to ensure that trainees meet competency standards in delivering patient care. However, a comprehensive summary of the literature on clinical assessment in postgraduate dental education is largely absent. Filling this gap is essential for developing effective assessment processes to help support competency‐based education at the postgraduate level. To address this gap, this scoping review aims to map the published literature on the assessment of clinical competence for postgraduate dental specialty trainees to identify knowledge gaps and future research areas.

**Methods:**

Guided by Arskey and O'Malley's framework, a comprehensive search was conducted across four databases (MEDLINE, EMBASE, SCOPUS and Google Scholar) from 2005 until March 2025. The search was focused on subheadings related to assessment and postgraduate dental specialty training. Two researchers independently screened the literature for eligibility using inclusion/exclusion criteria, extracted key data and analysed data thematically. The research report strategy followed the most recent PRISMA guidelines for scoping reviews.

**Results:**

Thirty‐three articles met the inclusion criteria, with almost equal distribution between Asia, the United States and Canada and Europe. The articles covered diverse aspects of assessment in postgraduate dental specialty training, such as individual assessment tools, WBA, assessment systems, EPA and assessment format. The number of published articles on this topic increased over fourfold in the 2015–2025 decade compared to the previous decade. Qualitatively, four themes were identified in the analysis: (1) assessment concept: the why, what and who? (2) methods and tools used in assessment; (3) challenges, opportunities and areas for future research and (4) users' perceptions of assessment.

**Conclusion:**

Research on clinical competence assessment in postgraduate dental education is currently limited, particularly in terms of synthesis of assessment information for making progression decisions on individual trainees. Future research should focus on assessment systems that align with competency‐based education principles, leverage digital enhancements and are contextually relevant. This review underscores the complexities involved in designing and implementing a competency‐based assessment system within a clinical context and the need for user involvement and feedback to improve the effective utility of assessments and ensure engagement of all stakeholders.

## 1 Introduction

The adoption of competency‐based education has redefined curricula in health professions in recent decades [1, 2, 3]. Explicitly outlining the observable performance indicators that trainees must achieve to be considered competent provides a framework for assessment and progression of trainees throughout their training. In order to become qualified health professionals, trainees must not only meet minimum criteria of competence, but also demonstrate a commitment to lifelong learning, ongoing development and the pursuit of excellence in providing high‐quality patient care in challenging, complex and dynamic clinical contexts [4]. To ensure that trainees achieve these goals, assessment is viewed as a vital part of the learning process. How trainees are assessed determines what and how they learn; hence, assessment should be a strategic tool for improving teaching and learning in higher education [5]. Since it determines trainees' learning progression towards competence, assessment must be efficient, effective and tailored to the needs of trainees, institutions, the public at large and health care systems [6].

Evidence in the literature focusing on assessment in postgraduate dental specialty training and what kind of problems and needs educators and institutions have in supporting their decisions about competence has been limited. Although the literature about assessment within medical specialty training is well‐established, there remains a lack of information concerning assessment for measuring the development of postgraduate dental specialty trainees. Furthermore, studies on assessment in dental education have mostly been focused on the undergraduate level [3, 7, 8]. Though the emphasis on developing critical thinking skills, professionalism and communication skills continues from undergraduate to postgraduate transition. However, the needs of postgraduate dental specialty trainees differ from those of undergraduate students. Specialty training promotes progressive independence [9] which enables autonomous clinical practice. Moreover, specialty trainees are expected to have a deep understanding of dental concepts and techniques, as well as the ability to apply them in complex clinical scenarios requiring interdisciplinary collaboration. Additionally, postgraduate dental specialty trainings are considerably variable and context‐dependent, necessitating multifaceted assessment strategies that are tailored to the level of the learner and reflect the complexity and context‐dependency of the specialty context.

Similar to other health professions education, accreditation standards for dental specialty training programmes use attributes of high‐performing professionals after graduation as a metric for the efficacy of educational programmes. For institutions to meet these standards, there must be a clear assessment plan to ensure that all dental specialty trainees have met these standards via assessment and are competent at the specialist level.

### 1.1 Study Aims and Research Questions

The purpose of this scoping review is to summarise and evaluate the studies that have looked at assessment methods employed or reported in postgraduate dental specialty training. Postgraduate dental specialty training involves advanced education and clinical practice pursued by dentists following obtaining their initial dental degree, aimed at achieving specialisation in distinct areas of dentistry. This includes dental specialty training/residency, advanced dental training and their equivalence worldwide. The objectives of this review are to identify the published literature on assessment of clinical competencies in postgraduate dental specialty training and to gain insights into the experiences of both trainees and their supervisors regarding dental assessment. Finally, the review aims to make recommendations on the evidence gaps for future research.

## 2 Methods

A stepwise methodological framework as defined by Arksey and O'Malley [10] and advanced by Levac et al. [11] informed this scoping review. To further establish the methodological integrity of this review, the Preferred Reporting Items for Systematic reviews and Meta‐Analyses extension for Scoping Reviews (PRISMA‐ScR) guidelines were employed to facilitate research preparation and reporting [12]. The scoping review protocol was registered in the Open Science Framework (https://osf.io/4vq7b). A scoping review was considered an appropriate research method to navigate broad topics that may involve various study designs and methods, or to explore an area that has not been comprehensively researched [10]. Scoping reviews use systematic ‘mapping’ to explore and summarise the breadth and depth of the evidence, to identify the knowledge gap and to inform future research [11, 13]. This scoping review will contribute to the understanding of postgraduate dental specialty assessment and provide insights for the future development and implementation of competency‐based assessment strategies.

### 2.1 Stage 1: Identifying the Research Questions

In line with the objectives of this scoping review, the research questions were defined as follows:

1. What does the current literature report regarding clinical assessment for postgraduate dental specialty trainees?
2. What does the literature tell us about the experience of postgraduate dental specialty trainees and their clinical supervisors of assessment during their clinical training?

**FIGURE 1:**
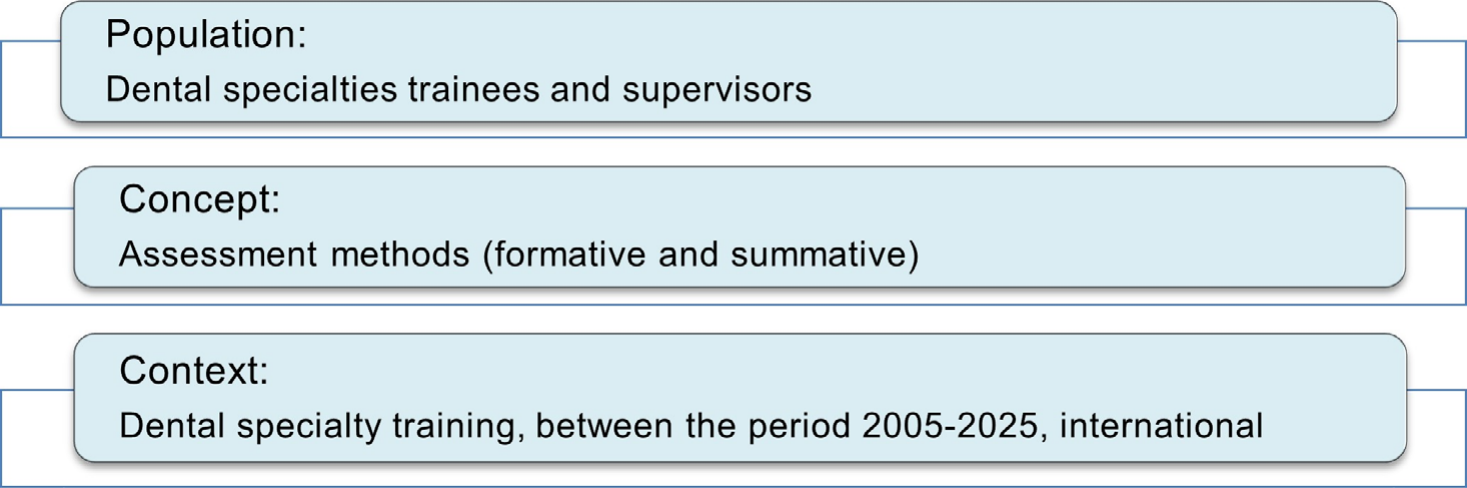
PCC mnemonic.

The questions and the eligibility criteria were designed on the Population, Concept and Context (PCC) elements using the guidelines recommended by the Joanna Briggs Institute (JBI) (Figure 1) [13]. The questions were designed to be as inclusive as possible without any restriction by assessment method in order to collect as much relevant data as feasible. When the questions about the concept of assessment as the topic of interest are placed within the context and population of the enquiry, they become specific. The context included the research publications between January 2005 and March 2025 and published in English in any postgraduate dental specialty training setting internationally.

The second research question of this study (as above) aimed to determine what is already known about how clinical supervisors and trainees experience assessment. This knowledge and understanding of individuals' perspectives offer valuable insights into the potential design of future assessment experiences in clinical settings, with the aim of improving engagement and perceived educational outcomes.

### 2.2 Stage 2: Identifying Relevant Studies

Using an iterative approach and with the guidance of an experienced medical librarian, the literature search strategy was developed. Combinations of the following search terms were used: assessment, evaluation, clinical competence, postgraduate dental education, graduate dental education, postgraduate dent*, graduate dent*, dental resident*, dental specialty. Table 1 provides a list of key terms searched and the Boolean search expression.

**TABLE 1:** Search terms.

Concept search terms	Boolean	People and context search terms
Searched with ‘OR’	‘AND’	Searched with ‘OR’
Assessment Evaluation		Postgraduate dental education
		Graduate dental education
		Postgraduate dent*
		Graduate dent*
		Dental resident*
		Dental specialty
		Clinical competence

A comprehensive search of the literature was performed using the following electronic databases: MEDLINE (via Ovid), Embase (via Ovid), Scopus and Google Scholar. As a first step, a limited search using search terms was conducted in MEDLINE. The search strategy was piloted to check whether the search terms and databases were suitable. Next, a second search was conducted across all databases to find relevant articles and was updated in March 2025. To supplement the database search, the reference lists of included articles for full‐text review were hand‐searched until no new article results appeared. The complete search strategies for all databases are included in Appendix S1. All records were imported from the databases to reference management software (EndNote X9, Philadelphia, PA, Clarivate Analytics).

### 2.3 Stage 3: Study Selection

Inclusion and exclusion criteria (Table 2) were determined prior to the start of the review. A selection protocol was devised to ensure that articles were selected consistently and provided information that was likely to address the research questions. To meet inclusion criteria, studies had to focus on assessments used in postgraduate dental specialty training/residency programmes.

**TABLE 2:** Selection criteria.

Inclusion criteria	Exclusion criteria
Peer‐reviewed journal article Conference papers Dental specialties trainees Published between 2005 and 2025 Full text available in English online	Undergraduate participants Grey literature Articles where population included undergraduate and postgraduate learners without providing separate data for each group Published before 2005 Unable to retrieve abstract or full‐text paper Written in another language than English

Assessments that captured various domains of professional competence such as procedural, behavioural (communication, professionalism), cognitive (decision making, diagnosis, self‐assessment) and clinical knowledge‐based competencies were included. Primary research, systematic reviews, meta‐analyses, qualitative and mixed‐method research, conference papers and narrative reviews published in peer‐reviewed journals that were available as full text were included. Articles that explained the piloting of an assessment tool, validation/reliability/feasibility testing of a proposed tool/assessment programme were also included.

To ensure that the study captures the most recent and pertinent changes in assessment practices related to competency‐based dental education in dental specialty training, our search was restricted to the past two decades, the period between January 2005 and March 2025. Letters to editors, commentaries, editorials, essays, study protocols, perspectives and opinion pieces were excluded. Undergraduate articles were excluded. Only those studies involving mixed populations (such as undergraduate and postgraduate dental learners) that provided postgraduate trainees' data separately were included. This also applies to reviews and consensus papers that cover assessment in dental education in general, without concentrating on postgraduate dental education specifically. Additionally, studies focusing on curriculum development without detailed elaboration on the assessment were excluded. Studies were excluded if a full text was unavailable in English or if a full text of the abstract did not exist (e.g., conference abstracts without subsequent full‐text publication).

After removing duplicates, the established inclusion and exclusion criteria were used to screen the articles by examining the titles and abstracts of identified studies independently by two authors (FAR and SP). If the information was inadequate to make a judgement, the full text was reviewed prior to the inclusion/exclusion decision. Then, inclusion/exclusion classification of the remaining articles was performed using the full text of all articles that met inclusion criteria in the first screening. Disagreements were resolved through discussion following each level of eligibility evaluation. The PRISMA Extension for Scoping Reviews (PRISMA‐ScR) checklist was used to document and report searches and screening, including the total number of sorted, selected, excluded and a final number of documents [14].

### 2.4 Stage 4: Charting the Data

This stage entailed extracting data from included studies. Two reviewers (FAR and SP) independently extracted the data from each included study. Data were gathered in Microsoft Excel (Microsoft Corporation, Redmond, WA). The research team designed the Excel data‐charting form to determine which variables to extract. Extracted data included: authorship, year of publication, country of study, study design, study setting, research question, study subjects, assessment method(s) or interventions (if any), key findings and recommendations and future research suggestions.

Given that the purpose of the scoping review was to map all existing research on assessment in dental specialty training, no article was excluded due to quality assessment [10, 11]. Scoping reviews favour including all the available literature since ‘critical appraisal would not be necessary if the purpose of the review is to … determine the nature and scope of existing literature’ [15]. However, the quality of the evidence was still examined and reported. The purpose of the quality assessment was not to include or exclude studies based on quality but rather to provide information on the methodological rigour of the relevant literature. The quality of quantitative studies was assessed using the Medical Education Research Study Quality Instrument (MERSQI) [16] and the Mixed Methods Appraisal Tool (MMAT) [17] was utilised for assessing qualitative and mixed‐method study designs in the present scoping review. For non‐empirical papers such as reviews, conference reports and theoretical papers, no critical appraisal tool was used.

### 2.5 Stage 5: Collating, Summarising and Reporting the Results

Stage five involved three distinct steps recommended by Levac: [11] analysing the data, reporting results and applying meaning to the results. Data analysis included a descriptive numerical summary and a thematic analysis. At first, a descriptive numerical summary describing the characteristics of included studies was prepared, and then all data collected in stage 4 were summarised into themes. For this step, the six phases of thematic analysis described by Braun and Clarke [18] were employed to provide an overview of the breadth of the literature, which assisted in making sense of the different data to represent responses to the research questions and relate findings to the study objectives. The initial phase of thematic analysis included data familiarisation. Therefore, all the articles were read prior to the coding process to have a general understanding of the results presented in the included studies. The results were read a second time for coding guided by the research questions. The codes were subsequently grouped into themes and sub‐themes. The themes were identified at a semantic level, wherein analysis was conducted within the explicit meanings of the content without looking for anything beyond what was written [18]. The results from the studies are presented narratively, and findings are articulated through themes to identify gaps in the evidence.

### 2.6 Stage 6: Consultation Exercise

Consultation exercises were conducted with a medical education stakeholder from Mohammed Bin Rashid University of Medicine and Health Sciences and with a stakeholder in dental education from Queen's University Belfast. Stakeholders were requested to read the draft of the scoping review and offer feedback on the usefulness of our results to clinical education practice. Stakeholders provided suggestions for the writing, layout and presentation of results. Following consultation, some sub‐themes and the way they fit together were revised.

## 3 Results

### 3.1 Overview

**FIGURE 2:**
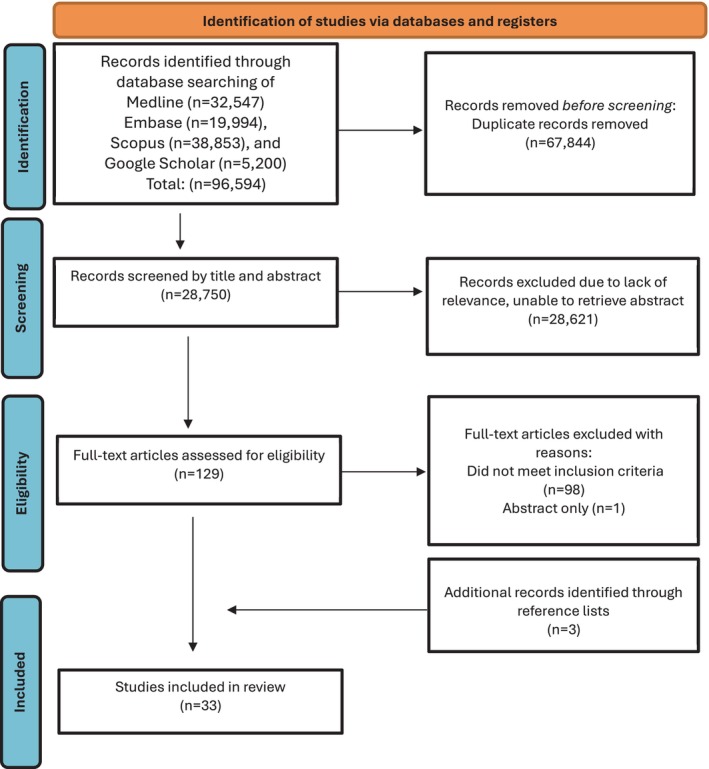
PRISMA‐ScR flowchart of the article search and selection stages.

**FIGURE 3:**
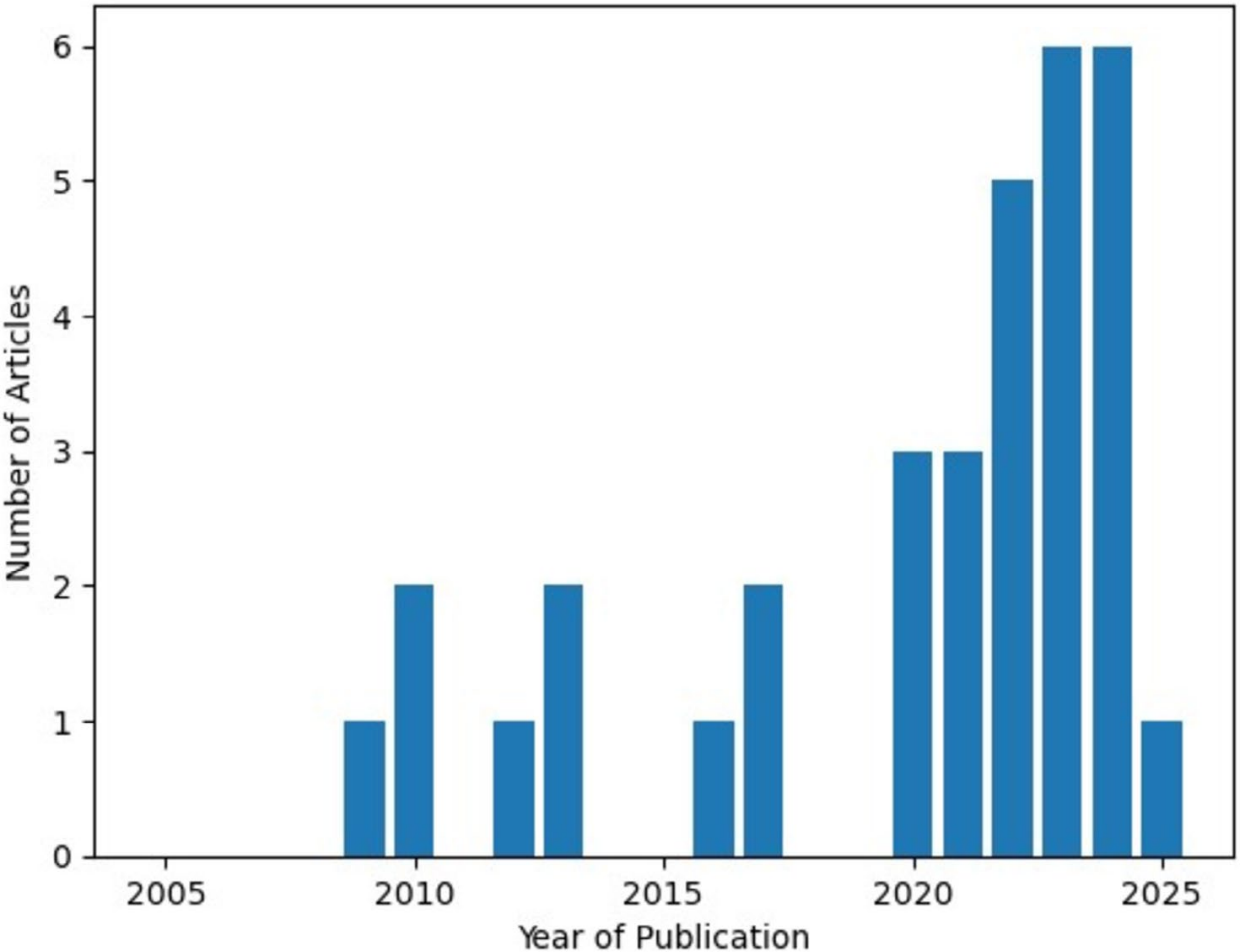
Article distribution according to the year of publication.

A total of 28 750 articles were screened for eligibility using the established inclusion and exclusion criteria. Of those, 33 full‐text studies were included in this review. Details outlining each phase of eligibility examination are illustrated in Figure 2. The included articles originated primarily from Asia (*n* = 11), the United States and Canada (*n* = 11) and Europe (*n* = 10), with one article from Africa (*n* = 1). The number of published articles on the topic under investigation has increased by over fourfold in the recent decade compared to the preceding decade (Figure 3). Summary demographics of included studies are outlined in Table 3. A full list of all 33 studies and additional detailed information is available in Appendix S2.

**TABLE 3:** Summary of article characteristics.

Characteristic	No. (%) of 33 studies	References
Decades of publication		
2005–2014	6/33 (18%)	[19, 20, 21, 22, 23, 24]
2015–2025	27/33 (82%)	[25, 26, 27, 28, 29, 30, 31, 32, 33, 34, 35, 36, 37, 38, 39, 40, 41, 42, 43, 44, 45, 46, 47, 48, 49, 50, 51]
Reported assessment methods		
Single tool (design, validity, acceptability, feasibility, effectiveness)	6/33 (18%)	[20, 25, 27, 28, 31, 34]
WBAs	7/33 (21%)	[22, 36, 37, 39, 48, 49, 50]
Assessment system	8/33 (24%)	[21, 23, 32, 34, 35, 40, 47, 48]
EPAs	11/33 (33%)	[29, 33, 36, 37, 38, 39, 41, 42, 44, 45, 46]
Assessment format	5/33 (15%)	[19, 26, 32, 33, 51]
Formative assessments (without specifying the tool or whether they are WBAs)	2/33 (6%)	[43, 45]
Summative assessment	5/33 (15%)	[34, 39, 43, 45, 49]
Location		
Europe	10/33 (30%)	[20, 22, 23, 24, 36, 37, 39, 43, 48, 49]
Asia	11/33 (33%)	[21, 27, 30, 31, 33, 35, 40, 41, 45, 47, 50]
The United States and Canada	11/33 (33%)	[19, 26, 28, 29, 32, 34, 38, 42, 44, 46, 51]
Africa	1/33 (3%)	[25]
Training specialty		
Implant dentistry	1/33 (3%)	[23]
Orthodontics	2/33 (6%)	[20, 47]
Oral and maxillofacial surgery	6/33 (18%)	[21, 26, 28, 33, 38, 45]
Orofacial pain	1/33 (3%)	[46]
Paediatric dentistry	2/33 (6%)	[29, 44]
Periodontology	5/33 (15%)	[27, 34, 39, 43, 49]
Prosthodontics	1/33 (3%)	[30]
Multiple disciplines	14/33 (42%)	[19, 22, 25, 31, 32, 35, 36, 37, 40, 41, 42, 48, 50, 51]

Overall, the included articles were predominantly quantitative studies (*n* = 12) [19, 25, 26, 27, 28, 29, 30, 31, 32, 33, 34, 35]. This was followed by literature review, both narrative and reflective, articles (*n* = 6) [20, 21, 36, 37, 38, 39], and mixed‐methods design (*n* = 6) [40, 41, 42, 43, 44, 45]. Descriptive studies that detailed the development of assessment tool/system, or the assessment in use, without incorporating quantitative or qualitative measurements, accounted for four articles (*n* = 4) [22, 46, 47, 48]. Additionally, there were three consensus reports (*n* = 3) [23, 24, 49], one qualitative study (*n* = 1) [50], and one conference report (*n* = 1) [51]. Quantitative study designs were utilised most frequently to measure validity, reliability and reproducibility of an assessment tool, cross‐sectional surveys and pre‐ and post‐training questionnaires assessing stakeholder‐identified outcomes or stakeholders' perceptions and satisfaction levels, to evaluate the quality and structure of the entrustable professional activities (EPAs). Whereas qualitative designs were used most often to explore perspectives of trainees and supervisors, while mixed methods designs were used either as the Delphi method for developing EPAs and surveys aimed at gaining a comprehensive understanding of postgraduate specialty training across various countries or universities, or to combine objective and quantitative data from assessment tools with subjective insights driven from users' feedback and satisfaction.

### 3.2 Critical Appraisal Within Sources of Evidence

The level of quality of the evidence varied from poor to excellent. A number of studies were poorly designed and imprecisely reported, making it difficult to draw conclusions. The quality score of quantitative articles averaged 10.35 points, ranging from a minimum score of 6 to a maximum score of 13.5 (maximum MERSQI total score is 18). Research questions were not clearly stated in one of the qualitative and mixed‐method studies [40]. A summary of MERSQI and MMAT quality appraisal is provided in Appendix S3.

Four overarching themes (each with a number of sub‐themes) were identified from the reviewed articles to answer the research questions, and these are explained below.

### 3.3 Theme 1: Assessment Concept: The Why, What and Who?

#### 3.3.1 Domains of Assessed Professional Competencies

The majority of the articles emphasised the requirement for mapping assessment to outcomes, establishing the competencies, or range of abilities that a trainee can demonstrate, to support competency‐based assessment [20, 24, 34, 35, 36, 37, 38, 42, 43, 47, 48, 49, 50, 52, 53, 54]. This alignment of assessment to predefined competencies under domains of competence was reported as a concept [23, 24, 49] and as an already established practice in some of the reviewed articles that explored competency‐based assessment [21, 22, 28, 38, 39, 41, 45, 48]. However, not all studies explicitly shared or referred to a competencies' framework utilised as the reference point of their proposed, piloted, or implemented assessments.

In one article [22], assessment systems were organised to measure competencies under four interconnected domains: clinical knowledge, communication, professionalism and managerial. The European consensus on assessment of implant dentistry competencies [23], emphasised the need for an assessment process to evaluate the three key components of clinical competence: knowledge, attitudes and skills. However, they did not elaborate on the domains of each competence. Similarly, Jolly [19] highlighted the use of eight evaluation topics (clinical skills, clinical judgement, diagnostic competence, level of independence, patient management, recordkeeping, response to faculty guidance and time management) that were not organised into a framework. Cully and Schwartz [29] surveyed paediatric dentistry residency programme directors (*n* = 57) in the US and Canada and reported an overlapping range of domains for postgraduate trainees' assessment including clinical competency, clinical skills, didactic knowledge, professionalism, ethics, time management, communication skills, research and leadership.

A few articles [22, 29, 44] referred to specialty‐specific competencies outlined by accreditation bodies, such as the Commission on Dental Accreditation (CODA) and General Dental Council (GDC) approved curricula for specialty training to guide assessment. Similarly, the Conference of Postgraduate Deans and Directors (COPDEND) in the UK has outlined postgraduate dental specialty training assessment for progression that reflects the assessment of GDC competence [48]. An article [35] designed an assessment system for postgraduate dental specialty training based on the American Dental Education Association (ADEA) core competencies for incoming general dentists. These competencies encompass critical thinking, professionalism, communication and interpersonal skills, health promotion, practice management and informatics and patient care. The authors asserted that the professional proficiency of dental clinicians, regardless of their stage, remains the same. In the 2024 consensus report by the European Federation in Periodontology (EFP), the competencies were systematically listed under nine categories [49]. Additionally, a number of studies referred to general domains of competence described by medical accreditation bodies, such as those developed by the Accreditation Council on Graduate Medical Education (ACGME) [patient care, medical knowledge, practice‐based learning, interpersonal and communication skills, professionalism and system‐based practice]. While one study [21] referred to ACGME without detailing the specialty‐specific competencies or how the general competencies are tailored or applied to the unique contexts of each dental specialty, another study [38] demonstrated how oral and maxillofacial surgery residency programmes can align with ACGME core competencies. In several studies, however, the competency framework employed or mapped to and the defined or assessed competencies were not explicitly specified or shared in published articles.

More recent articles advocate translating multiple competencies into competency domains within a predefined EPA framework to assist clinical supervisors in assessment and feedback provision based on trainees' level of training [29, 36, 37, 38, 41, 42, 44, 45, 46]. Hawkins et al. [46] used the knowledge, skills and attitudes (KSAs) encompassed within the domains of the Canadian Medical Educational Directives for Specialists (CanMEDS) competency framework to exemplify the development of EPAs in postgraduate orofacial pain programmes. Likewise, Carlson presented how the integration of EPAs is intended to help achieve the alignment between ACGME core competencies and specialty‐specific competencies, thereby bringing together the abstract nature of competency‐based training to clinical assessment [38]. Whereas, Younas et al. [41] suggested that their proposed EPAs for physician‐patient communication are not limited to a particular competency framework, hence allowing for potential linkage to different competency frameworks. Lastly, EPAs have been used to establish a competency framework for oral cancer management within the context of specialty training in maxillofacial surgery [45].

In numerous studies, the evaluation of different levels of Miller's pyramid of competence has been highlighted as a vital theoretical grounding [22, 23, 27, 39, 40, 49, 50], with particular emphasis on the importance of the assessment of clinical competence to address the highest level (‘does’ level) of the pyramid in the supervised workplace [22, 23, 37, 42, 50]. Kelly et al. [36] endorsed Ten Cate's [52] proposal of adding ‘trusted with future care’ as a fifth assessment level to Miller's pyramid for postgraduate dental assessment.

#### 3.3.2 Outcomes of Assessment

Of the research included in this scoping review, 12 studies explicitly reinforced the need for formative assessments to indicate progress towards competency and summative assessments to determine the achievement of competency [24, 30, 31, 33, 36, 37, 40, 43, 46, 47, 48, 50]. Some research solely discussed formative assessment's relevance in encouraging positive learner growth without incorporating it into the summative part of an assessment programme [25, 27, 30, 35]. It was also reported that assessment used to determine residents' graduation readiness varied from daily clinical progress/observation, faculty evaluation, written exam, time‐based evaluation and practical exam [29].

With the introduction of EPAs and entrustment decision making being a key component of EPAs, a number of studies [33, 34, 38, 39, 54] regarded the formative assessment (several observations from different faculty members) as a valuable tool for guiding trainees and providing evidence for making summative entrustment decisions to assess the training progression and determine the readiness of postgraduate dental trainees to practice independently. Two recent studies suggested that EPAs may also be used for self‐assessment purposes [37, 41]. None of the included studies reported on the impact of assessment on learning outcomes and patient care.

Lastly, one study [34] examined the relationship between an institution's specialty training outcomes and the In‐service Examination (AIE) administered by the American Academy of Periodontology (AAP) as a standardisation examination. This comprehensive multiple‐choice examination is aimed at allowing periodontology training programmes to evaluate curriculum content and provide trainees with a low‐stakes external assessment to benchmark their learning. However, this study found no correlation between AIE performance and the evaluated specialty training outcomes.

#### 3.3.3 Who Oversees Assessment Examinations?

In this scoping review, two studies elaborated on who is responsible for assessments in specialty training [43, 48]. The formative assessments were conducted by the university alone or in collaboration with hospitals, within practice by specialist supervisors, or with Royal Colleges of Surgeons in 24 out of 25 countries in the study on periodontology specialty in Europe. For summative assessments, in 11 out of 25 countries, universities were the sole entities responsible for conducting the assessments. The remaining universities engaged in a broader range of collaboration when it came to the summative decision, involving the government bodies, hospitals, the EFP, the specialist society, Royal Colleges of Surgeons, National Examination Boards and Ministry of Health [43]. In a recent overview article of the Gold guide for dental core and specialty training in the UK [48], the responsibility of reviewing assessments was regarded as the function of the review of the competency progression panel.

At the implementation and administration level, the US survey on the evaluation methods in postgraduate dental specialty training revealed that programme directors are highly involved in the assessment process. The programme directors underscored the need for additional support from both full‐time and part‐time faculty to nurture a shared responsibility towards assessment. Only 40% of 226 programmes included part‐time faculty participation, highlighting the necessity for improved support for part‐time faculty members to actively participate in the assessments [32].

### 3.4 Theme 2: Methods and Tools Used in the Assessment

#### 3.4.1 Assessment Tools and Assessment System

A broad range of assessment tools was either described or implemented in the included articles. Some of these assessment tools were derived from well‐established instruments in medical education, while others were created by integrating existing assessment tools used in medical education to develop a more concise instrument. Tools were grouped into two categories: Workplace‐Based Assessments (WBAs) and non‐Workplace‐Based Assessments (non‐WBAs) (Table 4).

**TABLE 4:** Summary of assessment tools.

Assessment tool group		Assessment tool	References
WBAs	Indirect clinical observation	Case‐based discussions (or presentations) (CBDs), case reviews	[22, 23, 39, 47, 48, 50]
		Logbook review/treatment progress report	[39, 47, 48]
		Structured written reflections, reflective essays	[39]
	2 Direct clinical performance observation (observational assessment) with feedback provision	Mini‐clinical evaluation exercise (mini‐ CEX)	[21, 22, 23, 27, 39, 40, 48, 50]
		Direct observation of procedural skills (DOPS)	[22, 23, 39, 40, 45, 48, 50]
		Procedure‐based assessments (PBAs)	[22]
		Structured clinical operative test (SCOT)	[23]
		Evaluation of clinical events (ECE)	[39]
		Unlabelled three‐question tool: supervision (Zwisch Scale), performance and difficulties using the SIMPL (System for Improving and Measuring Procedural Learning) application.	[26]
		Direct clinical observation and Zwisch feedback with real patients using communication checklist	[41]
	3 Feedback obtaining tools for assessment of non‐clinical aspects of clinical performance	Mini‐peer assessment tool (Mini‐PAT)	[22]
		360‐degree appraisals and multi‐source feedback (MSF)	[22, 23, 36, 39, 45, 49, 50]
		Patient assessment questionnaire (PAQ)/Patient satisfaction questionnaire (PSQ)	[22, 39]
		Team assessment of behaviour (TAB)	[22, 39]
Non‐WBAs	Written assessments	Annual written progress assessment at the end of years of training and at the end of training	[21, 34, 39, 43, 45, 47]
	2 Objective structured practical/clinical examinations (OSPE/OSCE)	Oral and Maxillofacial Objective Structured Assessment of Technical Skills (OMOSATS)/Objective Structured Assessment of Technical Skills (OSATS)	[28, 31]
		Other simulation‐based assessments	[21, 22, 25, 30, 39, 40, 41, 43, 45, 47, 49]
	3 Structured Viva or oral assessment (including several treated cases or unseen cases)		[20, 21, 43, 45, 47, 49, 50]

To assess the four levels of Miller's pyramid, various assessment methods were proposed in the included articles [22, 23, 36, 37, 39, 49, 50]. For the ‘know’ and ‘know how’ levels, written and/or oral exams were suggested to foster evidence‐based practice and develop a problem‐based approach. The ‘show how’ level was assessed in a simulation environment using computer‐based technologies and objective structured clinical examinations (OSCE). OSCE not only allows the assessment of the ability of the trainee to perform, diagnose and formulate a treatment plan but also observes the trainee's interactions with patients, empathy and communication skills. Finally, the assessment of the ‘does’ or ‘application’ level included WBAs [direct and indirect observation of clinical performance, multi‐source feedback (MSF)], EPAs and self‐reflection.

Of the 33 articles reviewed, 11 involved WBAs as individual sets of assessments, as part of a programmatic assessment system and/or portfolio content, or to highlight the shortcomings of WBAs [33, 34, 37, 43, 45, 46, 47, 48, 49, 50, 52]. Combining the information gathered by multiple assessment tools and by different supervisors was advocated as authentic assessment in these studies. Though individual tools were commonly discussed [20, 25, 27], several articles reported designing or piloting several individual assessment tools in different settings as part of programmatic assessment [21, 22, 23, 34, 40, 49], and two articles maximised the value of WBAs as part of EPA‐based competence assessment methods [37, 41, 45].

In addition, the number and frequency of formative and summative assessments remained variable, based primarily on anecdotal data. The number of required or suggested WBA or formative activities required varied between one and numerous events in a year‐long programme based on type of assessment [22, 32, 40, 50]. Without distinguishing between formative and summative assessments, the study by Jolly et al. found that the majority (58%) of US programme directors assess the performance of their postgraduate dental trainees semi‐annually, while 23% and 20% do so daily and quarterly, respectively [19]. In a recent survey conducted in the US and Canada, 56.1% of paediatric dentistry programme directors used formative assessment twice a year, whereas 96.5% administered summative assessments twice yearly [29]. In another recent survey of US specialty training programmes, most conducted assessments twice or four times a year, with assessment frequencies ranging from daily to annually. Programme directors believed that the frequency of assessment could be improved. The Gold guide for dental core and specialty training in the UK does not specify the quantity or frequency of formative competence assessments [48]. This flexibility allows training programmes to tailor their assessment schedules based on their specific needs and contexts.

The majority of research investigating validity, reliability, feasibility, usefulness, suitability and effectiveness concluded that the relevant instrument and methods were valid, reliable, feasible and effective for their intended purpose [20, 21, 25, 26, 30, 38, 39, 52, 55, 56]. Reliability was invariably assessed using the coefficient of Cronbach's alpha to assess the internal consistency and inter‐rater agreement [30, 31]. The face and construct validity, as well as the improvement in trainees' scores when compared over time, were used to support the validity and effectiveness of the reported assessment method(s) [27, 28, 30, 31]. The validity and reliability evidence for the recently developed EPAs in postgraduate dental education were established by linking them to the existing WBAs and providing a comprehensive description of the robust development process to verify that EPAs accurately represent the work of a specialty [36, 37, 41, 42]. Moreover, the quality and structure of the EPAs were evaluated using the Equal rubric as a standardised method to assess various dimensions of the EPAs, including clarity, specificity, observability and measurability [33, 37].

The feasibility, usefulness, effectiveness and suitability of assessment tools and methods were mainly examined by collecting survey feedback, whether it be quantitative or qualitative, from expert panel, trainees and/or supervisors to investigate acceptability and perceived learning value [25, 27, 28, 30, 31, 35, 40]. Kaban et al. [26] examined the feasibility of their assessment method [System for Improving and Measuring Procedural Learning (SIMPL)] and trainee and supervisor engagement by evaluating the number of performed tasks, time to complete assessments, elapsed time to submission, proportion of assessments with narratives feedback and trainees' response rates.

The consensus report on assessment methods in periodontology training programmes in Europe recommended incorporating computer‐based technologies (such as virtual reality and augmented reality tools) and OSCE for clinical competence assessments [49]. In a recent survey that investigated specialist training in periodontology in Europe [43], it was found that in all 25 countries, more than one method of summative assessment was employed to determine the competence of graduates and their readiness to qualify as a specialist. These methods included written examination (15 countries), oral examination (20 countries), OSCE (9 countries) and portfolio (22 countries). In another survey of EFP‐recognised programmes, clinical competence was primarily assessed through a combination of direct observation in the clinic, use of simulated environments and case portfolios [39]. In contrast, Yang et al. [35] demonstrated that the structure of the summative assessments as part of the comprehensive assessment system (CAS) in China comprises eight stations of OSCEs.

#### 3.4.2 Assessment Rating Method

Rating method is the format in which the tool captures performance information. In this review, ten studies described a variety of scoring and rating scales to assess behaviour or performance [21, 22, 26, 27, 28, 30, 31, 36, 42, 46]. The most frequently used item scaling mechanism was ordinal Likert global rating scales ranging from 5‐ to 10‐point scales with category descriptors [22, 26, 27, 30, 31]. Checklists are another form of rating mechanism included in the tools examined in this review [41]. They serve to assess the trainee's performance against a predetermined list of criteria. One study [28] combined task‐specific checklist and global rating scale (Table 5).

**TABLE 5:** Description of rating methods reported in the included studies.

Assessment tool	Scale with descriptors	References
CBD	1–6 scale: 1,2 = Needs improvement, 3 = Borderline, 4 = Acceptable, 5, 6 = Above expectations	[22]
mini‐CEX	9‐point scale: 1, 2, 3 = Unsatisfactory or Need Improvement or to be strengthen, 4, 5, 6 = Satisfactory or up to standard, 7, 8, 9 = Superior or excellent	[27, 30]
SIMPL	A combination of Zwisch 4‐level scale (level 1 = show and tell; level 2 = active help; level 3 = passive help; level 4 = supervision only) and performance 4‐level scale indicating the readiness for independent practice (1 = unprepared; 2 = inexperienced with the procedure; 3 = intermediate performance; 4 = practice ready performance; and 5 = exceptional performance)	[26]
Communication specific checklist	No details were reported	[41]
OSCE	5‐point scale: the details of the five points were not reported	[30]
OMOSATS/OSATS	Combined task‐specific checklist as (‘done correctly’ or ‘not done/incorrect’) [28] and (‘not done’, ‘done but not completely’, ‘done completely’) [31], and global rating scales (1–5 with description for 1,3,and 5) for not task‐specific items	[28, 31]

Innovations in rating methods that were recommended or implemented in the most recent reviewed articles included the entrustment‐based supervision scale [36, 37, 41, 42, 45, 46]. Entrustment scales use levels 1–5 to quantify the degree to which a trainee is regarded as safe to undertake a certain task or procedure independently. They consist of the following: the trainee 1. May observe, cannot perform the task, 2. can perform under direct supervision, 3. can perform with indirect supervision, 4. can perform the task independently, 5. is able to supervise others doing the task. For the postgraduate trainee to be entrusted with an EPA, they must demonstrate that they are competent in unsupervised practice (level 4), which entails attaining specific competence [42]. Hawkins et al. [46] stressed that supervisors must be periodically calibrated to ensure that assessment and feedback are relevant to the learner's level.

#### 3.4.3 Portfolio

Studies in the scoping review advocated the use of assessment portfolios to gather evidence and record progress and achievement. Several studies suggested the utility of portfolios in supporting assessment for learning by providing feedback to trainees and assessing their development over time [21, 22, 23, 35, 39, 43, 48]. Portfolio was regarded as one of the information sources for the end of training assessment for periodontology in 22 countries in Europe [43]. Suggested components of a portfolio in these studies included a personal development plan, trainee's self‐assessment and reflective journal, logbook of treated cases/procedural experiences, case‐based reports and presentations, work‐based competency assessment forms, patient satisfaction surveys/MSF/mini‐peer assessment tool (Mini‐PAT)/360 degree, assessments of clinical audits, assessment of teaching, publications, scores of written tests and OSCE [21, 22, 23, 35, 43, 48]. However, besides the use of assessment portfolios to gather evidence, except for three articles [21, 39, 48], most studies did not provide a practical way to make progression or graduation decisions. Kadagad and Kotrashetti [21] suggested the use of a global rating scale to evaluate portfolio entries for postgraduates in oral and maxillofacial surgery, followed by the conversion of those ratings into percentages and classification of those percentages into four categories (good = 80% and above, satisfactory = 65% and above, borderline = 50% and above, not satisfactory = less than 50%). Byrne et al. [48] in their overview of the Gold guide in the UK, described the use of the online Intercollegiate Surgical Curriculum Programme (ISCP) as a source of evidence for the review of competency progression panel to ensure the achievement of requisite competencies by specialty trainees. Goldstein et al. [39] indicated that clinical cases portfolios require annual assessment by training programmes for progression and assessment by the EFP examination board for certification. To earn the EFP certificate of completion of specialised training in periodontology, submission of five fully documented clinical cases covering various facets of periodontal and implant therapy is necessary. However, the provided information did not contain specifications about the scoring or qualitative evaluation of portfolios throughout the decision‐making process used by the panel to ground their summative progression or graduation decision.

#### 3.4.4 Assessment Format

A number of studies reported on the format used for assessment. Two studies documented and recorded assessment data in local contexts using paper‐based forms, including feedback forms [22, 30]. In 2012, a research study revealed that all accredited U.S. dental specialty programme directors supported the transition to a computer‐based format [19]. In 2022, the American Dental Education Association (ADEA) summit addressed the use of an electronic assessment application and the MyEvaluations tool for monitoring competency assessments, documenting the progress of dental specialty trainees and calibrating faculty [51]. A recent survey in CODA‐accredited postgraduate dental specialty training programmes found that 70.6% of these programmes use electronic means for assessment, predominantly via desktop computers and mobile devices. New Innovations, MedHub and Qualtrics were among the most frequently used software programmes [32]. The same study reported that the use of mobile devices for assessments was associated with higher levels of satisfaction among programme directors [32]. Additionally, Byrne et al. [48] reported the use of e‐portfolio (ISCP) for specialty training in the UK. Chen et al. [33] presented the convenience and effectiveness of Emyway in Taiwan for implementing EPAs, reporting good satisfaction levels among both trainees and supervisors regarding its application. Finally, one study piloted data collection using a mobile application called SIMPL to assess operative skills performance of oral and maxillofacial surgery trainees [26]. Operationalisation and challenges of computer‐ and web‐based tools, as well as the use of e‐portfolios for data storage, learning analytics and progress decision making, were not adequately reported.

### 3.5 Theme 3: Challenges, Opportunities and Identifying Areas for Future Research

#### 3.5.1 Challenges and Opportunities

One study raised the concern that in some dental postgraduate assessment systems the emphasis remains on recording numbers, e.g., in the form of logbooks, rather than assessing competencies [29]. Hence, competency is typically dependent on quantitative assessment (i.e., attaining a particular number of cases), not qualitative assessment of specialty‐specific competencies (i.e., critical thinking, treatment planning, readiness for independent practice, etc.) [29]. A number of postgraduate programmes used assessment criteria created and implemented by the institution [19, 29, 47, 50]. Cully and Schwartz [29] showed that without standardised graduation requirements, 65% (36 of 56) of US paediatric dentistry programme directors were concerned that not all dental residents graduate with the same baseline level of competency [29]. Some studies highlighted the overarching role of governing bodies, accreditation and professional organisations (e.g., COPDEND and CODA) in providing a competency framework and a uniform formal outline for implementation, monitoring and quality evaluation of assessment consistency [19, 22, 29, 44, 48]. However, the assessment of trainees' expected competence to become independent care providers is a subject of dispute among different postgraduate dental programmes.

The collaborative effort of educators from many institutions to communicate about their assessment systems and develop bridges to identify common practices is a strength. Several studies aimed to collect the opinions of programme directors of dental specialty programmes, as well as educational and clinical expertise at national levels, employing survey and the Delphi method [19, 29, 32, 33, 42]. Another study compared the assessment methods as part of Orthodontics curriculum between top institutions across the world using checklist surveys [47]. Further, there were opportunities for experts around the world to share experience and thoughts on assessment through workshops, conferences and Delphi process, which resulted in the production of consensus guides for designing assessment in certain fields of dentistry [23, 41, 45, 46, 51]. The 2022 ADEA summit paper underscored the significance of discipline‐specific benchmarking in dental specialty training, accentuating its role in enhancing the evaluation of trainees' preparedness and progress, which in turn enhances educational outcomes [51]. In the same vein, Eaton et al. [43] in 2022 proposed further harmonisation of competences for newly qualified specialty trainees in periodontology across Europe, a notion that was subsequently reflected in the published work of Herrera et al. and Goldstein et al. [39, 49] While the collaborative effort to unify training outcomes in periodontology is commendable, further international collaboration is imperative to comprehensively map the competencies and competence domains in other dental specialties. This will help ensure the service quality and patient safety. Yet, the possibility of variability in the implementation of a structured competency‐based learning and assessment system across different programmes and specialties should also not be overlooked.

WBAs were deemed a valuable assessment method for measuring competency by assessing postgraduate trainees' performance in actual clinical dental practice [22, 30, 40, 48]. However, studies also reported that WBA implementation requires a substantial investment of resources. First, to increase interrater reliability, to understand the learning value of formative assessments and to engage in the assessment process, ongoing comprehensive training for supervisors is required [22, 50]. Second, to ensure reliability, the number of assessments and assessors would need to be high enough, which may be unfeasible in certain settings [40]. Lastly, other common logistical challenges to implementing WBA, such as time restrictions in the clinic, the need for a large patient pool, increased workload for supervisors with conflicting responsibilities and the difficulty of translating concepts of competency assessment into clinical parameters, were reported in the studies [30, 36, 50]. The average time commitment required to complete mini‐CEX per encounter varied from 29 to 45 min for observation and 10 to 15 min for feedback in two different studies [27, 30].

A key element of assessment systems is the use of multiple assessment points to inform summative decisions [22, 36, 41, 48]. However, papers rarely explained the interface and interdependence between formative assessment and summative assessment. Recent research identified EPAs as an opportunity to standardise the evaluation of postgraduate dental trainees' clinical competency. In addition, the existing WBA methodologies may be utilised to assess EPAs because of their observational and authentic nature [36]. Yet, in postgraduate dental education, there is no clear consensus on clinical competency for independent practice [36]. Despite the recommendation of the possibility and suitability of using EPAs for competency‐based assessment in postgraduate dental education and a call for its adaptation [29, 36, 37, 38], only four studies [41, 42, 44, 46] put out a proposal for an EPA framework for postgraduate dental training. Hawkins et al. [46] and Cully et al. [44] have proposed EPA frameworks for orofacial pain residency and paediatric dentistry programmes, respectively. Meanwhile, Ramaswamy et al. [42] have suggested handoff EPAs as a shared competency in both undergraduate and specialised dental programmes, and Younas et al. [41] have developed EPAs for physician‐patient communication for dental and medical postgraduate training programmes. Chen et al. [33] proposed, piloted and validated three levels of tooth extraction EPAs for different stages of dental training from undergraduate years to oral and maxillofacial surgery specialty training. Moreover, the 2024 review by Goldstein on specialist training programmes in periodontology suggests the definition of specific EPAs as a potentially valuable approach for effectively linking the professional tasks to their respective mini‐curricula and WBAs [39]. In the same review [39], survey results indicated that only one EFP programme utilised EPAs for competence assessment. However, despite the expanding body of literature on EPAs, a cross‐sectional survey of paediatric dentistry programme directors in the US revealed that over two‐thirds of respondents were unaware of the use of EPAs to promote trainees' independence [29].

#### 3.5.2 Identifying Areas of Future Research

Studies on assessment tools and methods, particularly WBAs and EPAs, consistently highlighted the necessity for further research on a larger scale (i.e., larger sample sizes and across multiple institutions) to collect data on the effect of feedback on clinical training outcomes such as learning, clinical skills and improved patient care [22, 31, 33, 35, 50]. Emphasis was also placed on the necessity of conducting longitudinal studies to evaluate the long‐term effectiveness and impact of the formative and summative assessments on the clinical competence and professional development of dental specialty trainees [31, 38]. Moreover, studies called for further investigation into how postgraduate dental institutions are implementing WBAs [22, 30, 50], how frequent and how many WBAs throughout the year need to be conducted to increase validity and reliability and how formative assessment informs summative decision [22]. Additionally, future research should take into account the perspectives of trainees on improving the assessment system, including their needs, preferences, desired frequency of assessments and feedback, preferred methods of feedback and areas where they require more feedback [32].

Studies identified the need for more understanding of the full potential and application of portfolio in making progression decisions to inform summative assessment [23]. The findings also imply that patients, peers and staff members can serve as reliable assessors [35, 36, 49, 50]. However, how their accumulated data may be employed at a summative level for decisions related to competency attainment was not discussed and is an important part of competency‐based assessment that demands more study.

Due to the paucity of research on EPAs in competency‐based dental education and given their success in medicine, studies on EPAs in dentistry called for more effective research on formulation, validation and calibration to support implementation and assessment of EPAs as an important area for future research. This would require a national or international consensus on competence, development of core competencies and EPAs for independent clinical practice for each discipline [29, 36, 37, 38]. Furthermore, five articles stated that EPAs can potentially improve objectivity, increase the standardisation of assessment criteria and eventually may help in reducing the risk of observational bias [29, 36, 37, 44, 46]. Further implementation research would provide a deeper insight into subjectivity in entrustment decision making using EPAs in everyday authentic clinical practice.

### 3.6 Theme 4: Trainees' and Clinical Supervisors' Perceptions of Assessment

Eight studies investigated supervisors' and/or trainees' views of a tool or assessment system and the perceived learning impact to understand how supervisors and trainees experience assessment. One study used a qualitative approach [50], seven studies took a quantitative survey approach [25, 27, 30, 31, 32, 33, 40], and one study combined quantitative questionnaires with qualitative free‐response sections [19]. For the most part, trainees and their supervisors were satisfied and felt that the assessment had a positive effect on trainees' learning. There was a general agreement between both groups' perspectives on assessment; however, trainees placed particular emphasis on supervisors' engagement with assessment, the need for more explicit assessment criteria and improved utilisation of feedback. Conversely, supervisors expressed concerns about increased workload and time constraints within the clinical setting.

#### 3.6.1 Perceived Learning Value

Numerous facets of formative assessment were seen positively by most supervisors and trainees in several studies [27, 30, 40, 50] According to the trainees in the reviewed studies, assessment contributes to the knowledge, attitudes and skills development, provided them several learning opportunities to realise their weaknesses [50], boosted their confidence [27] and lowered their examination anxiety [27]. In the study by Rawekar [40], trainees and supervisors found formative assessment helpful in improving trainees' self‐assessment. Similarly, 73.8% of 226 postgraduate specialty programmes in the US indicated that trainees are provided with an opportunity for self‐assessment.

In addition, one study revealed that trainees attributed the learning value of the assessments to the complexity of the clinical cases, the supervisors' active involvement in the assessment process and the consistency of the assessment process rather than to the assessment instruments themselves [50].

One study found a significant consensus among supervisors and trainees on the usefulness of CBD (and sometimes, the most useful tool) as an assessment for learning and feedback provision tool [50]. Finally, two studies surveyed postgraduate dental specialty trainees about OSCE and OSATS implementation. Trainees found OSCE and OSATS methods to be adequate measures of clinical or technical skill, supporting their inclusion in practical course assessments [25, 31]. Additionally, trainees recommended incorporating OSCE into postgraduate training modules for better familiarisation [25].

#### 3.6.2 Consensus on Assessment Criteria and Minimum Standard Setting

In the calibration, criteria and rubrics context, the study by Amir Rad et al. [50] showed trainees' concerns about different subjective expectations, vagueness of assessment criteria and various interpretations of performance. The supervisors in this study also recognised that calibrating their assessment and standardising the method is quite challenging. The recommended solution was that expectations should be explicit and assessment rubrics should be clearer and better tailored to the clinical task. Nevertheless, it is tedious to create a checklist for each and every procedure [40].

#### 3.6.3 Supervisor and Trainee Training on Assessment

Another commonly highlighted comment was the importance of comprehensive training in the assessment use for both supervisors and trainees [50]. Surprisingly, in one study [50], some supervisors stated that they had received no formal training in the use of clinical assessment tools. Supervisors were concerned that WBA tools and systems demanded a substantial amount of their time and effort. A few supervisors viewed the documentation of continuing assessments as an extra burden [29, 50].

#### 3.6.4 Feedback

Trainees appreciated and welcomed feedback from supervisors and found this exercise valuable to gain insight into their development needs, knowledge gaps, or areas of skills and attitudes for improvement [30, 50]. However, some postgraduate dental specialty trainees struggled to associate the feedback with the clinical context [50]. Trainees indicated that feedback was sometimes found to be overly vague and of poor quality. Trainees valued feedback that contained specific suggestions for improvement in the form of written comments or real‐time verbal feedback when performing clinical tasks [30, 50].

The majority of postgraduate specialty training programmes in the US reported a strong correlation between the regular face‐to‐face feedback meetings and the perceived satisfaction of trainees with the feedback they receive [32]. Participants in Amir Rad's study [50] preferred narrative comments over numerical grades. Expert groups, dental specialty supervisors and trainees concurred that feedback from peers, staff and patients in the form of MSF, 360‐degree appraisal, or patient surveys heightened awareness in the assessment of communication skills and professionalism [35, 50]. Due to the variety of possible responses, trainees and their supervisors suggested the exclusively formative rather than summative use of this instrument [35, 50].

#### 3.6.5 User Satisfaction with New Assessment Implementation

Three studies [30, 31, 33] explored the satisfaction levels of trainees and their supervisors regarding newly implemented assessment methods and/or formats. Satisfaction was attributed to the smooth integration of new methods with existing clinical competence objectives, the ability to cover a wide range of diverse cases, ease of use, clarity of the assessment method and format, time efficiency and the provision of ongoing training and support.

## 4 Discussion

The aim of this scoping review was to explore the existing research relating to assessments used in dental specialty training and the experience of assessment among users. This review included 33 articles, which reported on a range of different specialties and represented a variety of publication types across different regions. Although these articles do not represent the entirety of the evidence regarding clinical assessment in dental specialties training, they serve as a representative sample of the literature that informs this topic and provides a basis for identifying several key patterns that can guide the practice and future research for those involved in the development, implementation and evaluation of clinical assessments.

In relation to the first research question examining the nature and range of literature that exists exploring assessment in postgraduate dental specialty training programmes, several articles mapped the assessment strategy to the learning outcomes using Miller's pyramid to aid in identifying the aim of assessment and selecting the most suitable assessment methods for a particular purpose [22, 23, 27, 39, 40, 49, 50]. This review found that the choice of appropriate assessment tools depends on the identification of what should be assessed and what the clear objective of each assessment is. Once the assessment purposes have been specified, postgraduate dental training programmes can then identify which assessment methods may be suitable and evaluate their utility to ensure they are using the best available assessments (fitness for purpose) [53, 54]. In the absence of a clear objective, assessments lack substantive meaning and are rendered futile for making informed competency decisions. Therefore, implicit in achieving valid quality assessments is the necessity to define the assessment's purpose [55].

Furthermore, the reported assessment approaches in this scoping review emphasised that establishing a minimum level of professional competence, while integrating the outcomes of contemporary assessments with the clinical contexts of trainees, remains a huge challenge. As such, there is a need for a greater contextualisation to measure trainees' function in practice to ensure obtaining a consistent ultimate assessment outcome, namely independent safe practice. Accordingly, there are authors who have highlighted this gap and advocate the use of EPAs as an appropriate tool for the assessment of postgraduate dental trainees [29, 36, 38, 42, 46]. Intuitively, curriculum and assessment are so intertwined that the effective implementation of one would require modifications to the other [57]. In other words, a clinical workplace curriculum with an EPA structure needs to be developed to map an EPA‐based assessment system. Currently, however, there is little evidence of EPAs' practical application in postgraduate dental specialty training. With the expansion and updates in dental specialty fields, there has been a significant focus on updating curricula, and to a lesser extent, the assessment systems, to ensure the attainment of learning outcomes. This is particularly evident in the systematically published and updated specialty training in periodontology from 2010 to 2024 [24, 49]. There are significant disparities in educational practices, patient care approaches, resources, including faculty expertise, infrastructure and technology, which might affect the adoption of standardised outcomes. In the absence of research on clinical competence assessment in postgraduate dental specialty training, institutions may rely on information from a different context to inform assessment. This issue, if not managed with caution, may lead to solutions that are not responsive to the context‐specific requirements. Hence, it is vital that research on clinical competence assessment from other health professions domains and regions be contextualised and scrutinised to extract the transferable lessons for designing and implementing an assessment system.

Given the number of articles included in this review stressing that the assessment's reliability and validity and effectiveness in demonstrating trainees' progress are dependent on the number and diversity of assessments, encounters and assessors across different contexts (triangulation of information assessing different aspects) [54], this review underscores the need for a better understanding of the optimal balance between the number of assessments and the increased likelihood of achieving clinical competencies [20, 22, 23, 40, 50]. Although few studies [22, 23, 30, 35] proposed the minimum number of observations or assessments necessary for deciding on attaining a competency, there was insufficient evidence for this review to determine whether these experiences are sufficient to certify that the trainee can perform these procedures independently under unknown conditions. Additionally, it is not surprising that competencies are intertwined such that there is an ongoing opportunity for multiple structured observations to assess competence domains such as professionalism, communication and cognitive competence (i.e., critiquing, reflection, reasoning, integrating knowledge to practice, etc.) in clinical competency assessment, which can be overlooked if the focus is on procedural tasks and the number of tasks completed. This review demonstrates the necessity to evaluate trainees often and thoroughly to collect sufficient data to provide a comprehensive and complete picture of their competence [56]. Thus, one area for further work in dental postgraduate education is research aimed at further understanding how gathered data is compiled and analysed and how the whole assessment system is closely regulated to ensure that all elements of competence are covered over time.

On the continuum of learning and assessment, the notion of programmatic assessment system entails the collection of evidence to confirm judgements about the trainee's development, progress and, ultimately, certification [58, 59]. Formative and summative assessments are essential for monitoring the development of a novice dentist into a competent specialist. A limited number of studies linked the summative assessment with the formative assessments. Leveraging formative assessment to detect patterns, track progress and enable early interventions among ‘at‐risk’ trainees would be beneficial [60]. Indeed, it can be argued that without holistic information of formative performance of the trainee, the summative function would be ineffective. Assessing a trainee's progress through a multi‐year training programme and across different clinical competence domains can reveal the level of proficiency at which the trainee is ready to practice safely and independently. This review revealed a dearth of data on holistic and longitudinal assessment of trainee performance and advancement decisions at postgraduate dental training level. This review makes it clear that in postgraduate dental specialty training, there is little literature on the aggregation of assessment information to make progression decisions on individual trainees, notwithstanding the sporadic efforts to improve the robustness of information offered by individual formative assessment events. The ability to assess all the dimensions that trainees are expected to demonstrate at the conclusion of each transitional level and at the graduation exit point is a formidable challenge. Logically, the number of assessment sources should be commensurate with the stakes of the assessment decision [61]. No study investigated how supervisors provide summative judgement on trainees' collective performance and what is considered adequate information to make such high‐stakes decisions. To create a comprehensive specialty training assessment strategy, it is necessary to incorporate a variety of assessment methods, encourage collaboration between universities and other governing or specialty entities, implement continuous feedback and promote reflective practices to foster holistic development [49]. This approach ensures that ‘the whole is greater than the sum of the parts,’ as it leverages the synergy of consolidated assessments to provide a more insightful and effective evaluation than individual assessments alone.

Considering the paucity of research on the assessment of postgraduate dental specialty trainees in general, and the assessment of graduates' readiness to enter unsupervised practice in particular, the time seems right for the development of a competency‐based assessment and progression system that is theoretically grounded and easily measurable in the daily clinical context. A framework comprising both general and specialised EPAs, as well as competency‐ and milestones‐based assessment, would likely result in a transition of focus from assessing trainees' ability to their capacity to provide patient care and professional outcomes. The implementation of competency‐based assessment requires a cultural shift towards regular assessments and feedback, which can be best facilitated by digital technology. Effective data collection should focus on enhancing the training by providing information to support the development of competence and to inform decisions about trainees.

In relation to the second research question, examining the experience of postgraduate dental trainees and supervisors' experience and perceptions of postgraduate assessment, this review identified designing, developing, implementing and evaluating a competency‐based assessment system as a complex process since it is driven by individuals within a multifaceted clinical context. Almost all the studies included in this review emphasised that the authentic challenge of any clinical competence assessment is in its integration into daily activities in the clinical environment with competing demands. The results of this review indicate that although WBA is a commonly endorsed/applied assessment method in dentistry, it faces challenges related to perceived time demands, users' insufficient assessment literacy and ambiguity surrounding its summative use. These dimensions have multiple interdependencies. Therefore, to successfully incorporate assessment methods into clinical practice, both trainees and supervisors need to collaborate. From this lens, having user buy‐in and feedback when developing and implementing an assessment programme is a key strategy for improving utility and engagement. Pilot testing and making iterative improvements informed by user feedback would help in refining the assessment system and increasing user satisfaction. Similar recommendations have been made by authors who developed an assessment strategy for competency‐based medical education [62] and other authors who explored solutions to address deficiencies in WBA implementation [63, 64, 65, 66]. Further research is needed to identify and understand the factors contributing to the limited participation and engagement of trainees and their supervisors in the assessment process, as well as to evaluate the educational impact of WBA on the development of clinical competence and the improvement of trainees' clinical performance.

The review found that training the supervisors and trainees is essential for any assessment system to be successful. Preparing the supervisors is necessary to ensure that they properly understand the assessment tools, the assessment results and how they relate to the other components of an assessment system. To achieve this goal when planning any assessment system, faculty development activities must be planned and given continuously to help supervisors build a shared mental model around the functions and goals of assessment [67]. Training trainees and supervisors in receiving and delivering high‐quality feedback using evidence‐based methods is a further important skill identified in this review that requires attention while designing a clinical assessment system. Similarly, training the trainees about the assessment framework and philosophy can influence their attitudes by promoting their agency in initiating the assessment and enhancing their sense of responsibility towards their learning [68].

A number of studies addressed the perceived value of feedback provision by trainees as part of formative and summative assessments and the quality of the feedback offered. Again, users' attitudes to learning benefits of assessment determine their view of assessment as a burden or as an opportunity for frequent observation and feedback. Trusting relationships in the clinical learning environment are key to such interactions, where learning gaps are viewed as opportunities for growth rather than failures [69]. Leveraging feedback, trainees and their supervisors should engage in a continuous, bidirectional dialogue to promote self‐assessment and, subsequently, self‐directed‐learning [70]. There is scope to further investigate the integration of learning theories and assessment in the dental workplace as a continuum by promoting feedback‐seeking and reflection behaviour and by encouraging trainees to gradually embrace greater responsibilities as they develop and progress towards becoming specialists. The context for learning and assessment is created by the social, cultural and physical environment [71] and this concept has broad potential for investigation in postgraduate dental training to promote an assessment system that is based on an assessment philosophy rooted in users' social culture and interactions. In addition, this review disclosed a dearth of evidence on the type (verbal vs. written) and quality of feedback provided to trainees, particularly those underperforming, to support their learning in postgraduate dental clinical settings through assessment.

The use of a well‐structured, customised, user‐friendly digital platform for efficient data entry, retrieval and analysis is essential for conducting meaningful longitudinal multi‐dimensional assessments. Multiuser platforms facilitate collaboration and communication among stakeholders (i.e., regulatory and professional entities, institutions, trainees and supervisors), while maintaining the security of assessment data. Although a number of studies [19, 26, 30, 32, 33, 48, 51] in this scoping review highlighted the potential of digitalising clinical assessments to reduce workload and enhance data management, the particular facets of exploring the challenges of developing and utilising computer‐ and web‐based tools to create e‐portfolios for keeping records, showcasing learning analytics and facilitating progress decision‐making have not been reported in dental specialties assessment literature. This gap suggests a need for further research to investigate these areas comprehensively and provide practical insights for efficient digital integration in clinical dental specialty education.

Finally, with regard to evaluation instruments, there was little data on the usefulness of the rating scales. In order to optimise the effectiveness and learning value of assessments as a means of repeatedly assessing performance against predetermined standards, a substantial level of collaboration is necessary between supervisors and trainees. Another challenge in re‐designing the assessment system that involves WBAs and EPAs is the emphasis on objectivity in dental specialty articles included in this review, which is intended to enhance the standardisation of assessment criteria. Nonetheless, the medical education literature has emphasised the opportunity to embrace the subjectivity in WBAs. This is because preformulated rubrics do not always align with the clinical supervisors' thoughts and observations in the dynamic, complex authentic clinical context. Consequently, the supervisors inevitably incorporate subjective judgements in their assessments [72, 73, 74, 75, 76]. Therefore, subjectivity seems unavoidable, or rather integral, to the structure of WBA. Although assessor training can improve consistency among assessors, there is a strength in combining objective and subjective judgements to make the assessment contextually relevant and meaningful [74, 77]. Possibly, additional research is required to comprehend how supervisors and trainees interpret the assessment scale and how effectively it provides a clear picture of what the trainee can perform independently and safely.

### 4.1 Strengths and Limitations

This is the first scoping review to focus on examining the literature on assessment in postgraduate dental education. Given the rapid adoption of postgraduate competency‐based educational approaches, this review may serve as a starting point for what is known, with further research and in‐depth studies expanding on this foundation.

Despite the systematic process followed in this scoping review, some limitations must be pointed out. A first limitation is that this review was limited to the English language, which led to missing relevant papers published in other languages. A second limitation is the selection of the literature search terms. This could be caused by the differing terminology used when referring to methods of assessment. For example, MSF is often not labelled as an ‘assessment’ or ‘evaluation’, even when it is used as part of formative assessment. Despite the efforts to make the search as thorough as possible, the study is limited to the articles uncovered in the four searched literature databases. A wider search using more databases might have generated additional references. However, even with the present search of four databases, multiple duplicate results were seen. Given the breadth of methodologies and specialties represented, this scoping review allows for an initial mapping of some of the contexts, tools, terms and perhaps conceptualisations of competency assessment present in the postgraduate dental education literature.

In accordance with the scoping review approach, despite the identification of methodological limitations pertaining to research instruments, they were included in this review. Finally, the low number of undesirable impacts or negative perceptions recorded in the studies may be the result of reporting or publication bias [78].

## 5 Conclusions

This scoping review of the available evidence on clinical assessment in postgraduate dental specialty training provides multiple insights. First, these findings are a valuable resource for educators involved in the assessment of specialty training in dentistry to reflect on their current practices. Second, research gaps were identified for future studies to focus especially on the need for more robust evidence on assessment systems that are context‐based and socially embedded in the institution's culture and are aligned with the principles of competency‐based education. The promotion of EPAs as an assessment tool underscores the interconnectedness of curriculum and assessment. Third, the results can inform stakeholders, institutions and accreditation organisations about the need for collaborative work and communication regarding the development of multifaceted programmatic assessment systems and workplace‐based entrustable decision‐making for postgraduate dental specialty training programmes.

## Conflicts of Interest

The authors declare no conflicts of interest.

## Supporting information


**Appendix S1:** Complete search strategy.

**Appendix S2:** Description of included articles.

**Appendix S3:** Quality assessment of included articles.

## Data Availability

The data that support the findings of this study are available on request from the corresponding author.
