# Supplementary material for: Assessing Clinical Competence of Postgraduate Dental Specialty Trainees: A Scoping Review
Source: Eur J Dent Educ. 2025 Oct 23;30(3):1073–91. doi: 10.1111/eje.70060 (PMC13383372; doi:10.1111/eje.70060)
Supplement: Supplementary file 1 — Appendix S1: Complete search strategy. [file EJE-30-1073-s001.docx]

**Appendix S1- Complete Search Strategy**

1. Database: Ovid MEDLINE(R) ALL,1946 to March 21, 2025

Search Strategy:

| 1 | Education, Dental, Graduate/ or postgraduate dental education.mp. or Clinical Competence/ (113992) |
| --- | --- |
| 2 | postgraduate dent*.mp./ (319) |
| 3 | graduate dent*.mp./ (202) |
| 4 | dental residen*.mp./ (269) |
| 5 | dental specialty.mp./ (281) |
| 6 | assessment.mp./ (1904879) |
| 7 | evaluation.mp./ (2099540) |
| 8 | 1 or 2 or 3 or 4 or 5/ (114698) |
| 9 | 7 or 6/ (3684616) |
| 10 | 8 and 9/ (39618) |
| 11 | limit 9 to (english language and yr="2005-Current")/ (32547) |

1. Database: Embase, 1974 to March 21, 2025

Search Strategy:

| 1 | postgraduate dental education.mp. or clinical competence/ (72388) |
| --- | --- |
| 2 | graduate dental education.mp./ (58) |
| 3 | postgraduate dent*.mp./ (314) |
| 4 | graduate dent*.mp./ (206) |
| 5 | dental residen*.mp./ (273) |
| 6 | dental specialty.mp./ (283) |
| 7 | assessment.mp./ (4058345) |
| 8 | evaluation.mp./ (2785637) |
| 9 | 1 or 2 or 3 or 4 or 5 or 6/ (73243) |
| 10 | 7 or 8/ (6195287) |
| 11 | 9 and 10/ (23886) |
|  | limit 9 to (english language and yr="2005 - Current")/ (19994) |

1. Database: Scopus

Search Strategy:

|  | (assessment OR evaluation) AND ("postgraduate dental education” OR "graduate dental education" OR "postgraduate dent*" OR "graduate dent*" OR "dental residen*" OR "dental specialty" OR “clinical competence”) |
| --- | --- |
| Limit to | Year 2005-2025  38853 |

1. Google Scholar

Search Strategy:

| With all the words  with at least one of the words | assessment evaluation  "postgraduate dental education" OR "graduate dental education" OR "dental residency" OR "dental specialty" |
| --- | --- |
| Custom range | 2005-2025 |
|  | 5200 [100 pages] |
