# Supplementary material for: Assessing Clinical Competence of Postgraduate Dental Specialty Trainees: A Scoping Review
Source: Eur J Dent Educ. 2025 Oct 23;30(3):1073–91. doi: 10.1111/eje.70060 (PMC13383372; doi:10.1111/eje.70060)
Supplement: Supplementary file 2 — Appendix S2: Description of included articles. [file EJE-30-1073-s002.docx]

Appendix S2- Data collection matrix of included 33 articles on assessment methods in dental postgraduate specialty training.

| **References** | **Country** | **Study design** | **Dental field/ specialty** | **Study Purpose or Question (if verbatim, use quotes)** | **Participants** | **Assessment methods (reported or intervention)** | **Brief summary of findings relevant to assessment** | **Future research suggestions relevant to assessment** |
| --- | --- | --- | --- | --- | --- | --- | --- | --- |
| Mattheos, N., et al. Assessment of knowledge and competencies related to implant dentistry in undergraduate and postgraduate university education. Eur J Dent Educ. 2009 | Europe  (European Consensus Workshop) | Consensus report | Undergraduate and postgraduate implant dentistry | “To investigate and describe a complete framework for the assessment of knowledge, competencies and attitudes related to Implant Dentistry.” | / | Postgraduate implant training and assessment portfolios may include five sections:  1- Personal development plan, 2- Student self-assessment and reflective practice, 3- Logbook of cases, 4- Case-based reports/presentations, 5 Competency assessment forms | / | More study is required to fully use portfolio in summative evaluation frameworks. |
| Van der Velden, U. and Sanz, M. Postgraduate periodontal education. Scope, competences, proficiencies and learning outcomes: Consensus report of the 1st European workshop on periodontal education – position paper 3 and consensus view 3. Eur J Dent Educ. 2010 | Europe  (the European Federation of Periodontology) | Consensus report | Periodontology specialty training | “The purpose of this article is to establish the scope, competences, proficiencies and learning outcomes in Postgraduate Periodontal Education aimed for the training of the modern specialist in Periodontology” | The European Federation of Periodontology (EFP) in collaboration with the Association for Dental Education in Europe (ADEE) | Assessment Requirements and Performance Criteria:   1. Clearly define and communicate learning outcome criteria to students. 2. Use multiple assessment methods and performance samples. 3. Employ both formative and summative assessments. 4. Ensure clear and demonstrable alignment of learning content, teaching methods, and assessments. | / | / |
| Cobourne MT. What's wrong with the traditional viva as a method of assessment in orthodontic education? J Orthod. 2010 | United Kingdom | Review | Orthodontics | / | / | Viva | Advantages: evaluates clinical reasoning, allows examiners to cover different subject areas, assesses knowledge, communication skills, mental agility, problem-solving and reasoning, clinical competence, and professionalism, is easy to organize and run, allows rapid throughput of large candidate volumes.  Disadvantage: Unstructured vivas are subjective and biased. | / |
| Jolly, P. A., et al. Evaluation of postgraduate dental specialty residents: a survey of program directors. J Dent Educ. 2012 | United States | Quantitative | Postgraduate dental specialty | “to identify methods currently used to evaluate residents’ clinical performance. Another objective was to determine if there were any patterns in terms of programs that were more likely to utilize electronic submission via email or computer application or less technologically advanced formats such as Scantron or other hardcopy forms in the evaluation of residents.” | U.S. postgraduate dental specialty program directors | / | 1- Hardcopy evaluations were the most prevalent means of submission.  2-Pogramme directors agreed that technologically improved submission modalities might improve efficiency. 3- Half of programme directors wanted to enhance their assessment procedure. 4- Programme directors who offered self-evaluation were more likely to state that their residents were satisfied with the feedback they provided. | Future research should include faculty evaluators to determine which methods they prefer and why, as well as to identify obstacles to modernization. Residents should also be polled to determine which suggestions they find most useful and appropriate. |
| Kalsi, H. K., et al. An explanation of workplace-based assessments in postgraduate dental training and a review of the current literature. Br Dent J. 2013 | United Kingdom | Descriptive | Postgraduate dental training | “The purpose of this article is to describe the use of WBAs currently available in dental postgraduate training and to explore their perceived value by trainees and trainers.” | / | WBAs | 1- Description of WBA instruments A- Observation of clinical encounters, B- Discussion of clinical cases: Case-based discussion (CbD), C- Performance feedback from clinical and non-clinical colleagues.  2- Weaknesses, strengths, and perceived value by trainees and trainers have been investigated to provide direction for the use of WBAs.  3-Using assessment programmes and an online portfolio. | Further research on 1- formative performance assessment to examine outcomes such as learning, implementation of clinical skills, and enhanced patient care.  2- how to actively use WBAs, such as how to provide and enhance the value of feedback, frequency, technique, and how to space out assessment over time. |
| Kadagad, P., & Kotrashetti, S. M. Portfolio: a comprehensive method of assessment for postgraduates in oral and maxillofacial surgery. J Maxillofac Oral Surg. 2013 | India | Review | Oral and maxillofacial surgery | / | / | Assessment programme for OMS | In the context of oral and maxillofacial surgery postgraduate programs in India, there is a paucity of information on the various methods of assessment that are suitable. Multiple assessment methods should be utilized so that the results can be triangulated to determine their validity and reliability. In addition to assessing competency at all levels of Miller's pyramid, assessment methods at the residency level should emphasize self-reflection, self-directed learning, and broader areas such as professionalism. | / |
| Omo, J.O. & Enabulele, J.E. Perception of postgraduate dental resident doctors towards the objective structured clinical examination. J Educ Ethics Dent 2016 | Nigeria | Quantitative | Postgraduate dental specialty | “The aim of the study was to assess the perception of postgraduate dental residents toward OSCE.” | Postgraduate dental residents | OSCE | Respondents had favourable opinions of OSCE, indicating that OSCE is acceptable to residents in residency training. | / |
| Kaban, L. B., et al. Evaluation of Oral and Maxillofacial Surgery Residents' Operative Skills: Feasibility and Engagement Study Using SIMPL Software for a Mobile Phone. J Oral Maxillofac Surg. 2017 | United States | Quantitative | Oral and maxillofacial surgery | “The purpose of this study was to determine the feasibility of using a smartphone application, SIMPL (System for Improving and Measuring Procedural Learning), developed by a multi-institutional research collaborative, to achieve a high rate of timely operative evaluations and resident communication and to collect performance data.” | Three faculty members, 3 senior residents. | SIMPLE smartphone application including 3 multiple-choice  questions: “1) What level of help (Zwisch Scale) was required by the trainee? 2) What was the level of performance? 3) How complex was the case?” | Participation of attending surgeons and residents was significant. The evaluation procedure was fast (evaluation could be completed in 2 minutes) and user-friendly, and it did not impose a significant burden on faculty and residents. Compared to the OMS department's previous paper-based feedback system, this evaluation instrument yielded significantly higher resident response rates (81%). | Future research will concentrate on the efficacy of the provided operative feedback, validating SIMPL as a tool for assessing technical competence, and determining how it influences the progression of technical skills from postgraduate year 1 to graduation. |
| Rathod, S. R., et al. Assessment of postgraduate dental students using mini-clinical examination tool in periodontology and implantology. J Indian Soc Periodontol. 2017 | India | Quantitative | Periodontology | “The aim of this study was to evaluate the feasibility and usefulness of the mini-CEX as an assessment and feedback tool in the postgraduate setting in periodontology.” | Eight postgraduate students and two assessors | Mini-CEX | The postgraduate students' medical interviewing, physical examination, professionalism and communication, counselling skills, clinical judgment, and organizational and efficiency skills improved over the course of four months of study.  The student's response to the mini-CEX was favourable. Prior to sensitization, the assessors were oblivious of any workplace assessment programme and consented to enhance the students' clinical competency learning. | Future research should concentrate: first, recognizing that there is no gold standard for assessing performance, and second, monitoring assessment rating quality. |
| Rawekar, A., et al. Formative assessment in practical for Indian postgraduates in health professions education: A strategic initiative towards competency-based education. J Family Med Prim Care. 2020 | India | Mixed methods | All the postgraduate departments of dental, medical, nursing | “The objectives of the study were to train and sensitize the faculty/supervisors and postgraduate trainees for formative assessment in practical as “Assessment for Learning” and to evaluate the programme for its feasibility and effectiveness.” | Postgraduate students of the first and second year and faculty supervisors of all the departments of dental, medical, nursing. | Observational Assessment tools used for formative were Mini-CEX and DOPS in addition to OSCE/OSPE. | Postgraduate scores in formative assessment showed improvement by time. Supervisors and students were satisfied with the formative assessments. Participants suggested feedback on how to improve the implementation of formative assessment. | / |
| Hawkins, J., et al. Entrustable Professional Activities in Postgraduate Orofacial Pain Programs. J Oral Facial Pain Headache. 2020 | United States | Descriptive | Orofacial pain (OFP) programme | / | Programme directors of all CODA-accredited Orofacial pain programmes | EPAs | development of 10 EPAs for OFP | / |
| Abtahi, M., et al. Comparison of the Postgraduate Orthodontics Curriculum in Mashhad Dental School with the Top Ten Dental Schools in the World. Iranian Journal of Orthodontics. 2020 | Iran | Descriptive | Orthodontics | “The aim of this study was to compare the orthodontics postgraduate curriculum of Mashhad School of Dentistry with the top ten dental schools in the world, with the intention of using the results to improve the quality of post- graduate orthodontic training.” | / | Written, oral, and practical test methods used for clinical competence assessment by different universities: Case evaluation, passing the written portion of the orthodontic board exam, chair-side feedback, on-the-job assessment, professional and ethical interactions with patients, professors, and staff. | An overview of evaluation of orthodontics residents at the top ten universities in comparison to the University of Mashhad | / |
| Amir Rad, F. A., et al. A qualitative study of trainer and trainee perceptions and experiences of clinical assessment in post-graduate dental training. Eur J Dent Educ. 2021 | United Arab Emirates | Qualitative | Postgraduate Dental Specialty | “The purpose of this study is to explore the perception of residents, faculty members and alumni concerning their experience with clinical assessment, and what configurations they consider as optimal to maximise the entailed learning experience.  The research questions:  1. How do trainers and trainees perceive WBA, and its effect on the learning experience?  2. What factors of clinical assessment tools need to be considered in designing context-specific WBA to maximise acceptance amongst dental post-graduate trainees and trainers?” | Second- and third- year residents in four post-graduate training programmes, recent graduates, and supervisors in HBMCDM. | Daily clinical logbook, Mini-CEX, DOPS, CBD, Oral examination | Two themes:   1. process: variables related to quality, workflow, and feedback. 2. people: variables related to the trainees and the trainers. | Future research should examine clinical assessment in CBE, in general, and in WBA across multiple institutions with varying characteristics, and possibly combine qualitative and quantitative data analyses. |
| Caminiti, M.F., et al. The Oral and Maxillofacial Objective Structured Assessment of Technical Skills (OMOSATS) examination: a pilot study. Int J Oral Maxillofac Surg. 2021 | Canada | Quantitative | Oral and maxillofacial surgery | “The aim of this study was to develop a simulated technical skills examination specific to oral and maxillofacial surgery, assessing the validity of the test and its psychometric properties and obtaining feedback on the examination from both resident trainees and expert evaluators.” | OMFS interns, PGY1,2,3, & 4, recently graduated residents, and current fellows were evaluated by blinded expert OMFS surgeons. | The Oral and Maxillofacial Objective Structured Assessment of Technical Skills (OMOSATS) examination. Tasks were evaluated using a validated global rating scale and task-specific checklists. | Strong face and construct validity for OMOSATS stations in determining training level based on demonstrated technical competence. | (1) OMOSATS examination development by adding new stations and establishing an examination bank.  (2) Further research evaluating a larger group is required. |
| Younas, A., et al. Entrustment in physician-patient communication: a modified Delphi study using the EPA approach. BMC Med Educ. 2021. | Pakistan | Mixed methods  (Delphi) | Postgraduate dental and medical specialties | “Our aim in this study was to develop EPAs for  physician patient communication by expert consensus,  along with their competencies, assessment strategies and  supervision levels focusing on the entrustment of patient  communication. Two research questions were developed according to our aims: 1. What are the desired EPAs for effective physician-patient communication? 2. What are the respective competencies (knowledge, skills, and attitudes),  assessment strategies and supervision levels needed for designing physician-patient communication EPAs?” | Content validation stage: 5 experts in medical education  The Delphi rounds: 27 experts | EPAs | Development of 4 EPAs for physician-patient communication | Examining the replication of this research in a different cultural environment to gain insights into the variations in the entrustment of communication-related responsibilities across different nations. |
| Kelly, G. M., et al. A literature review: Entrustable professional activities, an assessment tool for postgraduate dental training? J Dent. 2022 | United Kingdom | Review | Postgraduate dental specialty | / | / | EPAs | Description of EPAs, entrustment decisions, including entrustment supervision scales, and recommendations for the development of EPAs in dental curricula. | / |
| Cully, J.L. & Schwartz, S.B. Pediatric Dentistry Program Directors' Resident Assessment Techniques. Pediatr Dent. 2022 | United States | Quantitative | Postgraduate Pediatric dentistry | “The purpose of this study was to determine what methods pediatric dentistry program directors use to assess their residents for competency and readiness for graduation.” | Pediatric dentistry programme directors in the United States and Canada | Methods, domains, and frequency of formative and summative assessments | Observation/daily clinical progress drove most resident development to more complex procedures, clinical independence, and the trust to make clinical decisions. Most programme directors were interested in standardized parameters for determining competence but lacked confidence in the implementation's affordability and timeline. The majority of  program directors were unaware of EPAs. | To concentrate on the development of fundamental competencies and EPAs associated with the independent and safe practice of Pediatric dentistry. |
| Ramaswamy, V., et al. Entrustable  professional activities framework for  assessment of patient handoffs in  dentistry. J Dent Educ. 2022 | United States | Mixed methods  (Delphi) | Pre- and post-doctoral dental education | “The aim of this study is to report data on the lack of a proper patient handoff system in dentistry and dental education and to present a possible solution to integrate this into curriculum using the "EPA" framework.” | Delphi expert panel: volunteers from the 2017 ADEA Annual Session and targeted recruitment of Program Directors and Deans  Feedback from the 2019 ADEA  Commission on Change and Innovation  meeting | EPAs | The proposal of the D-PASS mnemonic as a trust-based assessment rubric for the handoff process entrustability of learners. | Application of D-PASS in other settings for further validation by assessing patient outcomes. |
| Eaton, K. A., et al. European Federation of Periodontology Survey of Postgraduate and Specialist Training in Europe in 2020. Eur J Dent Educ. 2022 | Europe  (the European Federation of Periodontology) | Mixed methods | Periodontology specialty training | “The aim of the survey was to establish which universities, and other educational establishments deliver postgraduate and specialist training in Periodontology in the 31 countries whose national periodontal societies/associations are members of the EFP and to obtain details of how these programmes are run, funded, regulated and evaluated.” | 31 national periodontal societies/ associations (members of the European Federation of Periodontology) | No method was specified for ‘during the training assessment’.  More than one method was used for summative (end of training) assessment: written examination, oral examination, OSCE, portfolio of treated cases, report of research, unseen cases. | Assessment during training: Conducted in 25 out of 29 countries, primarily by universities, sometimes with hospitals or specialist clinics.  End of training assessment: Present in all 25 responding countries, with universities often responsible, sometimes alongside government bodies or specialist societies. | The authors suggested: investigating ways to harmonize postgraduate and specialist training programs in Periodontology across Europe, exploring the effectiveness of different assessment methods, and confirming the competence of newly qualified specialists. |
| Niu, L., et al. A novel strategy combining Mini-CEX and OSCE to assess standardized training of professional postgraduates in department of prosthodontics.  BMC Med Educ. 2022 | China | Quantitative | Prosthodontics specialty  training | “This study aimed to explore whether combination of mini-CEX and OSCE represents a global-dimension assessment for postgraduate clinical competence in resident standardization training.” | 56 postgraduates in residency training from 2017 to 2019 | modified mini-CEX/OSCE feedback scales: 1- Mini-CEX assessing eight aspects of core competences in real patients, a 9-point rating scale organized in three levels. 2- OSCE to evaluate specific skills in vital tooth preparation in the simulation, assessing 6 aspects, a 5-point scale, with a total score of 30 points. (1) | 1. Enhanced Clinical Competence: The combined Mini-CEX/OSCE assessment significantly improved the clinical competence of postgraduate students in prosthodontics, with notable progress observed over the training period. 2. High Reliability: The evaluation system demonstrated high reliability, with Cronbach’s alpha values exceeding 0.7, indicating consistent and dependable assessment results.   Positive Feedback: Postgraduates reported high satisfaction with the training and assessment methods, appreciating the practical, objective, and fair evaluations along with real-time feedback.  These points highlight the effectiveness and reliability of the combined Mini-CEX/OSCE assessment strategy in enhancing the clinical skills of postgraduate dental trainees. | The study suggests future research should explore (1) additional evaluation methods like Case Study Teaching Method and Direct Observation of Procedural Skills (DOPS) to support various assessments in clinical education, (2) the integration of clinical practice with scientific research. Additionally, the development of a computerized dental clinical teaching and evaluation system is proposed to reduce examination workload, promote environmental protection through paperless processes. |
| Byrne, E., et al. The Gold guide for dental core and speciality training in the UK: a review. Br Dent J. 2023 | United Kingdom | Descriptive | Dental core training and speciality training | “This paper is set out to summarise the 2021 version of the Dental gold guide (the framework for dental core training and speciality training in UK) for a user-friendly reference for trainees and trainers.” | / | - 1. WBAs:   2. CBD   3. Clinical evaluation exercises   4. Direct observation of clinical skills | The Review of Competency Progression (RCP) facilitates the progress of specialty trainees in their training. The process involves evaluation of evidence of training progression by the RCP panel. The objective of this process is to ascertain that the required competencies are being acquired. Specialty trainees undergo an annual review of competency progression (ARCP).  To adequately prepare for the RCP, trainees must record and regularly update an online logbook to demonstrate their evidence of development. Work-based assessments are one of the types of evidence included in the trainees' logbook. | / |
| Cully, J. L., et al. Development of entrustable professional activities for post-doctorate pediatric dentistry education. J Dent Educ. 2023 | United States | Mixed methods  (Delphi) | Postgraduate Pediatric dentistry | “Since EPAs currently do not  exist for post-doctoral pediatric dentistry education, this study aimed to identify the essential tasks of the specialty and define corresponding EPAs using a modified Delphi method.” | The Delphi rounds: 11 Pediatric dentists. | EPAs | Development of 16 EPAs for Pediatric dentistry. | Once the field of pediatric dentistry explores the advancement of EPA in the future, it is essential to include a wider range of stakeholders to engage in this process. |
| Ehlinger, C., et al. Entrustable professional activities in dental education: a scoping review. Br Dent J. 2023. | France | Review | Under-graduate and postgraduate dental education | “We sought to explore the rationale and goals for developing EPAs in dentistry. We wished to identify the processes used for EPA development in dental education as well as the resulting EPAs (level of training, topics, specialties, etc).” | / | EPAs | Standardisation of assessment and fulfilment of societal expectations are the main benefits of EPAs in competency-based assessment.  The process of developing EPAs, EPA topics, EPA-related competence assessment, and entrustment scale were among the themes identified in this scoping review.  There is no evidence on the implementation of EPA-based assessment in postgraduate dental education. | Future research should examine the implementation of EPAs and the assessment of learner development across various dental specialties. |
| Al-Jewair, T., et al.  Resident selection, assessment, and management:  Proceedings of the 2022 ADEA Advanced Dental Education  Summit. J Dent Educ. 2023 | United States | Conference report | Advanced dental education residents | “The objective of the summit was to discuss best practices for selecting, assessing, and managing advanced education residents while highlighting emerging issues in advanced dental education.” | Attendance was from a broad range of faculty, including deans, department chairs, program directors, hospital administrators and faculty involved in advanced dental education, along with members from other American Dental Education Association Councils. | Two concepts:   1. Use of technology tools 2. Benchmarking | 1. Electronic assessment application and MyEvaluations for assessment of residents, which tracks competencies and provides aggregate data over different time periods   (2) The use of benchmarks or milestones to assess resident readiness and progress, which involves developing discipline-specific benchmarks. | Evaluate the effectiveness of electronic assessment tools like MyEvaluations in tracking resident progress and their impact on faculty calibration and competency-based curriculum assessment. research the development and implementation of discipline-specific benchmarks in advanced dental education to assess their impact on resident performance and program outcomes. |
| Maybodi, F. R., et al. Assessment of suturing and scaling skills of periodontology and oral medicine residents by OSATS method: a pilot study. BMC Med Educ. 2023 | Iran | Quantitative | Periodontology and oral medicine specialties | “(1) Was there an interrater agreement in the use of global rating scale and Checklist?  (2) Was there any difference in the obtained scores on the basis of demographic factors of the residents? Were the residents satisfied with the way it was held and also the outcome?” | 6 periodontology specialty trainees and 3 oral medicine specialty trainees | OSATS | The study found excellent inter-rater agreement for the checklist and the global rating scale, indicating high reliability. Higher scores were associated with more advanced years of education, while no significant gender differences were observed. Additionally, 88% of residents expressed satisfaction with the OSATS method, appreciating its ability to identify and improve their weaknesses. | 1. Future studies with larger sample sizes and across multiple centres to improve the generalizability of the findings. 2. Comparative evaluations of periodontics residents and undergraduate students or periodontal specialists to enhance understanding of skill development and assessment. 3. Implement longitudinal studies to monitor the progression and enhancement of residents' skills over time utilizing the OSATS method. 4. Investigation of broader application of the OSATS method to evaluate additional practical skills in dentistry beyond suturing and scaling, to confirm its relevance across diverse dental procedures. |
| Ringer, J. B., et al. An overview of current evaluation methods of postgraduate  dental specialty residents. J Dent Educ. 2023 | United States | Quantitative | Postgraduate dental specialty education | “This research sought to discover the present techniques and protocols utilized for evaluating the performance of dental residents.” | 226 program directors of postgraduate dental specialties accredited by the Commission on Dental Accreditation (CODA) | Qualitative and quantitative assessments and utilization of electronic applications or software | 1. Evaluation Frequency: Most programs conduct evaluations twice or four times a year. 2. Participants: Program directors and full-time faculty are heavily involved; part-time faculty less so. 3. Types of Evaluations: Both qualitative (comments) and quantitative (scores) methods are used. 4. Submission Methods: Evaluations are submitted electronically, often using software like New Innovations, MedHub, and Qualtrics. 5. Feedback Delivery: Feedback is primarily given face-to-face. 6. Self-Evaluation: Residents often have opportunities for self-evaluation. 7. Program Satisfaction: Most program directors are satisfied, but improvements are needed in faculty participation, resident feedback, and software navigation. | Future research should (1) survey residents to identify specific aspects of evaluation needing improvement. (2) to gather common perspectives on the desired frequency and methods of feedback, as well as the performance qualities residents prioritize. Additional research is required to clarify the potential factors that may be contributing to the observed lack of participation, if applicable. |
| Carlson, E. R. Entrustable Professional Activities in Oral and Maxillofacial Surgery Education: A Faculty Development  Construct. J Oral Maxillofac Surg. 2024 | United States | Review | Oral and maxillofacial surgery | It is the purpose of this article to review the concept of EPAs that represent a hopeful solution to the theoretical and abstract nature of exclusive competency-based training assessments in resident education. | / | EPAs | 1. Traditional competency-based assessments in oral and maxillofacial surgery are often subjective and lack meaningful feedback. 2. Entrustable Professional Activities (EPAs) are proposed as a solution, providing specific tasks that can be entrusted to trainees once they demonstrate competence. 3. The study emphasizes the need for robust faculty development to effectively implement EPAs and improve resident assessments. 4. A list of 13 EPAs was developed, intersecting with ACGME core competencies, to enhance the training and evaluation process. 5. Faculty and resident engagement is crucial for the successful adoption of EPAs. 6. EPAs aim to provide more objective and meaningful evaluations, enhancing the overall assessment process. | The article suggests future research in:   1. Faculty Training: Assess the effectiveness of faculty development programs for EPAs. 2. Resident Engagement: Enhance strategies to boost resident motivation and engagement. 3. EPA Impact: Evaluate the effects of EPAs on resident performance and patient outcomes. 4. Assessment Tools: Develop and validate specific tools for EPA evaluation. 5. Longitudinal Studies: Track long-term outcomes of residents trained with EPAs. 6. Inter-professional Collaboration: Study the role of teamwork in implementing EPAs effectively. |
| Chen, J. L., et al. Developing competency-based medical education for dental education in Taiwan: A pilot study of tooth extraction entrustable professional activities. J Dent Sci. 2024 | Taiwan | Quantitative | Undergraduate, postgraduate, and oral and maxillofacial surgery specialty training. | This study aimed to evaluate the initial implementation of CBME by focusing on a specific EPA within Taiwan dental education. | The participants were 17 students from undergraduates, postgraduates, and Oral and maxillofacial surgery, and 7 clinical teachers. | EPAs | 1. EPA Development: Three levels of tooth extraction EPAs were created for different stages of dental training (UGY, PGY, OS-R) and validated using the Delphi method. 2. Assessment Tools: Separate assessment forms for students and teachers were designed, covering various competencies and criteria. 3. Implementation Platform: The Emyway platform was used for data collection, analysis, and feedback, facilitating the assessment process. 4. Satisfaction Levels: Both teachers and students reported moderate satisfaction with the EPA design and usage, with no significant differences between their experiences. 5. Quality EPAs: The EQual rubric scores for the EPAs were high across all levels, indicating strong consensus on their quality. | The study suggested expanding EPAs to other dental specialties and integrating CBME into Taiwan's dental education to align with international standards. |
| Kim, H. S., et al.  Predictors of standardized in-service examination  performance and residency outcomes in a graduate  periodontics program. J Dent Educ. 2024 | United States | Quantitative | Periodontology specialty | “The aims of this single-centre retrospective observational study were to assess the influence of learner- and education-related factors on In-service Examination (AIE) performance and determine whether AIE scores predict objective residency outcomes related to knowledge in the field of periodontology and general cognitive ability.” | In-service Examination (AIE) scores from 10 periodontics residency classes at a single centre | In-service Examination | 1. No educational or learner-related characteristic correlated with AIE performance, and although there is agreement that the AIE is a well-designed assessment tool, AIE performance did not correlate with any assessed residency result. 2. Trainees and programmes would be better served by raising the stakes of the AIE, motivating participants to prioritize AIE preparation. | 1. Comprehensive Study and Global Perspective: Conduct a detailed study to analyse AIE usage across all programs, gaining a global view to identify best practices and integration opportunities. 2. Expert Involvement and Optimization: Involve Health Professions Education (HPE) experts to optimize the stakes and standardization of the AIE and re-evaluate the necessity of offering two test formats. 3. Utilization of AAP Meetings: Use AAP annual meetings and workshops to discuss and share ideas about the AIE, facilitating immediate benefits and improvements. |
| Herrera, D., et al.  Consensus report of the second European Consensus  Workshop on Education in Periodontology. J Clin Periodontol. 2024 | Europe  (the European Federation of Periodontology) | Consensus report | Periodontology specialty | “To identify and propose changes necessary in periodontal education at three levels, namely undergraduate, specialist and continuing professional development (CPD), with respect to learning outcomes, competencies and methods of learning/training and evaluation.” | EFP workshop participants | written (including multiple-choice, single best  answer, short answers or essay-based) and/or oral exams, OSCE, WPBA | 1. Clearly defined criteria for the learning outcomes. 2. Multiple methods of assessments and multiple assessments of performance should be carried out. 3. Both formative and summative assessments should be used. 4. The assessment must be aligned to the learning and teaching. 5. Basing the assessment levels on Miller Pyramid: Written and/or oral exams for ‘knows’ and ‘knows how’ levels, OSCE for “show how” level, and WPBA for “does” level. 6. Emphasis on continuous formative assessment, feedback, and reflective learning | Further research is necessary to evaluate  the benefits of blended assessment for  knowledge and skills in periodontology. |
| Goldstein, M., et al.  Structure, governance and delivery of specialist training  programs in periodontology and implant dentistry. J Clin Periodontol. 2024 | Europe  (the European Federation of Periodontology) | Review | Periodontology specialty | “To update the competences and learning outcomes and their evaluation, educational methods and education quality assurance for the training of contemporary specialists in periodontology.” | / | Assessment of theoretical and clinical Competences:   1. Assessment of ‘know’ level: written (including   multiple-choice, single best answer [SBA], short answers or essay-based)  and/or oral exams.   1. Assessment of ‘know how’ level: Written (including multiple-choice, SBA, short answers or essay-based) and/or oral exams. 2. Assessment of ‘show how’: computer-based technologies, OSCE 3. Assessment of “does”: Workplace-based assessment (Observation of clinical performance, Discussion of clinical cases/case-based discussions, Multi-source feedback), Alternative assessments (EPAs), Self-reflection | 1. Clearly defined criteria for the learning outcomes. 2. Multiple methods of assessments and multiple assessments of performance should be carried out. 3. Both formative and summative assessments should be used. 4. The assessment must be aligned to the learning and teaching. | 1. In the   future, it might be useful to define clear and specific EPAs for periodontology  training, each connected to a mini-curriculum with its  own WPBA. |
| Hanif, M., et al. Designing Entrustable Professional Activities for Treatment  Planning of Oral Cancer by Maxillofacial Surgery Residents:  A Modified Delphi Study. Int J Dent. 2024 | Pakistan | Mixed methods  (Delphi) | Oral and maxillofacial surgery specialty | “The aim of this study was to develop a competency framework based on EPAs in oral cancer management by postgraduate trainees in oral and maxillofacial surgery through expert consensus.” | Content validation stage: 5 consultant maxillofacial surgeons  The Delphi rounds: 42 consultants oral and maxillofacial surgeons | EPAs | 1. EPAs Development: Five EPAs were identified, focusing on oral cancer management, based on 38 competencies refined through expert consensus. 2. Assessment Strategies: Proposed strategies include MCQs, SEQs, OSCEs, DOPS, 360-degree feedback, and portfolios. 3. Supervision Levels: Defined supervision levels for each EPA, ranging from direct supervision to independent practice. 4. Consensus and Validation: High content validity was achieved through expert feedback and iterative Delphi rounds. 5. Implementation: The EPA framework provides a structured roadmap for training supervisors to map learning outcomes in oral oncology for postgraduate trainees. | The proposed EPA framework must be tested across various training centres to assess its feasibility and utility in clinical educational environments, as well as its integration with other workplace-based assessment tools. Input from educational providers, trainees, and patients can be utilized to enhance and advance the proposed EPA framework. |
| Yang, J., et al. Delphi Study to Evaluate Competency Comprehensive  Assessment System for Professional Degree of Dentistry. J Dent Educ. 2025 | China | Quantitative | Professional master’s degree and specialty training | “In this study, we proposed to conduct an appraisal of Comprehensive Assessment System to further enhance the scientific rigor and feasibility of the assessments, as well as to refine the evaluation system, such as items, content, and methods through Delphi procedure.” | 40 experts from 13 universities in China | 1. Formative assessment: Portfolios [minimum procedural experiences, number and points of indicative operations, chairside procedure-based assessment, patient’s satisfaction survey, case report, case presentation], 2. Summative assessment: OSCE | 1. Through two rounds of Delphi surveys, consensus was achieved among 40 experts regarding the assessment content, criteria, and standards, indicating strong expert approval for the multidimensional approach of CAS. 2. The CAS integrates research assessment (dissertation) and clinical assessment (oral clinical proficiency), including both formative and summative assessments, to evaluate the clinical competency of dental students. 3. The study concluded that the CAS is acceptable and has the potential for further promotion, with most content highly agreed upon by experts, indicating its feasibility and scientific rigor. | Investigating practical implementation strategies for the chair-side procedure-based assessment, including the optimal timing and methods for conducting these assessments.  Expanding the application of CAS to other institutions and contexts to validate its generalizability and adaptability across different educational settings. |
