# Supplementary material for: Assessing Clinical Competence of Postgraduate Dental Specialty Trainees: A Scoping Review
Source: Eur J Dent Educ. 2025 Oct 23;30(3):1073–91. doi: 10.1111/eje.70060 (PMC13383372; doi:10.1111/eje.70060)
Supplement: Supplementary file 3 — Appendix S3: Quality assessment of included articles. [file EJE-30-1073-s003.docx]

Appendix S3- A. Quality assessment using the Medical Education Research Study Quality Instrument (MERSQI)

| Study | MERSQI for Quantitative Research | | | | | | | | | | |
| --- | --- | --- | --- | --- | --- | --- | --- | --- | --- | --- | --- |
|  | 1. Study design | 1. Sampling: Institutions | 3. Sampling: response of data rate | 4.Type | 5,6,7. Validity evidence for evaluation instrument scores | | | 1. Data analysis: sophistication | 1. Data analysis: appropriate | 10.Outcome | Overall score |
|  |  |  |  |  | Content | Internal  structure | Relationships  to other variables |  |  |  |  |
| Jolly et al. (2012) | 1 | 1.5 | 0.5 | 1 | - | - | - | 2 | 1 | 1 | 8 |
| Omo and Enabulele (2016) | 1 | 0.5 | 1.5 | 1 | 1 | - | - | 2 | 1 | 1 | 9 |
| Kaban et al. (2017) | 1 | 0.5 | 1.5 | 1 | 1 | - | - | 1 | 1 | 1 | 8 |
| Rathod et al. (2017) | 1 | 0.5 | 1.5 | 1 | - | - | - | 1 | - | 1 | 6 |
| Caminiti et al. (2021) | 1 | 0.5 | 1.5 | 3 | 1 | 1 | - | 2 | 1 | 1.5 | 12.5 |
| Cully and Schwartz (2022) | 1 | 1.5 | 1 | 1 | 1 | - | 1 | 2 | 1 | 1 | 10.5 |
| Niu et al. (2022) | 1.5 | 0.5 | 1.5 | 1 | 1 | 1 | 1 | 2 | 1 | 2 | 12.5 |
| Maybodi et al. (2023) | 1 | 0.5 | 1.5 | 3 | 1 | 1 | 1 | 2 | 1 | 1.5 | 13.5 |
| Ringer et al. (2023) | 1 | 1.5 | 0.5 | 1 | - | - | - | 2 | 1 | 1 | 8 |
| Chen et al. (2024) | 1 | 1.5 | 1.5 | 3 | 1 | 1 | - | 2 | 1 | 1 | 13 |
| Kim et al. (2024) | 2 | 0.5 | Not applicable | 3 | - | - | 1 | 2 | 1 | 1.5 | 11.99* |
| Yang et al. (2025) | 1 | 1.5 | 1.5 | 1 | 1 | Not applicable | Not applicable | 2 | 1 | 1 | 11.25* |

* adjusted accounting for “not applicable”.

Appendix S3- B. Quality assessment using the Mixed Methods Appraisal Tool (MMAT).

| Study | Methodological quality criteria | | | | | | | | | | | |
| --- | --- | --- | --- | --- | --- | --- | --- | --- | --- | --- | --- | --- |
|  | Screening questions  (for all types) | | 1. Qualitative study design | | | | | 1. Mixed methods study design | | | | |
|  | S1. Are there clear research questions? | S2. Do the collected data allow to address the research questions? | 1.1. Is the qualitative approach appropriate to answer the research question? | 1.2. Are the qualitative data collection methods adequate to address the research question? | 1.3. Are the findings adequately derived from the data? | 1.4. Is the interpretation of results sufficiently substantiated by data? | 1.5. Is there coherence between qualitative data sources, collection, analysis, and interpretation? | 2.1. Is there an adequate rationale for using a mixed methods design to address the research question? | 2.2. Are the different components of the study effectively integrated to answer the research question? | 2.3. Are the outputs of the integration of qualitative and quantitative components adequately interpreted? | 2.4. Are divergences and inconsistencies between quantitative and qualitative results adequately addressed? | 2.5. Do the different components of the study adhere to the quality criteria of each tradition of the methods involved? |
| Rawekar et al. (2020) | Can’t tell | Can’t tell | - | - | - | - | - | - | - | - | - | - |
| Amir Rad et al. (2021) | Yes | Yes | Yes | Yes | Yes | Yes | Yes | - | - | - | - | - |
| Younas et al. (2021) | Yes | Yes | - | - | - | - | - | Yes | Yes | Yes | Yes | Yes |
| Ramaswamy et al. (2022) | Yes | Yes | - | - | - | - | - | Yes | Yes | Yes | Yes | Yes |
| Eaton et al. (2022) | Yes | Yes | - | - | - | - | - | Yes | Yes | Yes | Can’t tell | Yes |
| Cully et al. (2023) | Yes | Yes | - | - | - | - | - | Yes | Yes | Yes | Yes | Yes |
| Hanif et al. (2024) | Yes | Yes | - | - | - | - | - | Yes | Yes | No | No | Can’t tell |
